# Evaluation of the synergistic and antagonistic antibacterial effects of pulsed electromagnetic fields combined with ciprofloxacin and nanochitosan

**DOI:** 10.1038/s41598-026-63235-2

**Published:** 2026-07-23

**Authors:** Alaa M. Khalil, Nevine L Seiffein, Wessam M. El-Refaie, Noha S. El-Salamouni, Hassan Abdulaal, Jannik Peters, Mai I. Elkaliouby

**Affiliations:** 1 Basic Sciences Department, Faculty of Engineering, Pharos University in Alexandria, Alexandria, 21544 Egypt; 2 Microbiology and Immunology Department, Faculty of Pharmacy, Pharos University in Alexandria, Alexandria, 21544 Egypt; 3 Pharmaceutics and Pharmaceutical Technology Department, Faculty of Pharmacy, Pharos University in Alexandria, Alexandria, 21544 Egypt; 4 School of Engineering Sciences, KTH Royal Institute of Technology, Stockholm, Sweden; 5 Physics and Chemistry Department, Faculty of Education, Alexandria University, Alexandria, 21544 Egypt

**Keywords:** Ciprofloxacin, Chitosan nanoparticles, Pulsed electromagnetic waves, Bio-electromagnetic interactions, *E. coli*, *P. aeruginosa*, *S. aureus* SDG 3: Good Health and Well-being, Biological techniques, Biophysics, Biotechnology, Microbiology, Nanoscience and technology

## Abstract

This study introduces an innovative electromagnetic nano-approach to combat high-severity bacterial infections without relying solely on high-dose antibiotics. We investigate the synergistic potential of extremely low-frequency pulsed electromagnetic wave (ELF PEMW) exposure (< 20 Hz) as a physical catalyst to enhance the bio-activity of ciprofloxacin-loaded chitosan nanoparticles (Cipro-C-NPs). This method provides a pivotal alternative for managing *Escherichia coli*, *Pseudomonas aeruginosa*, and *Staphylococcus aureus* in an era of escalating multi-drug resistance. Cipro-C-NPs were synthesized with high encapsulation efficiency. Bacteria were subjected to a multi-factorial screening involving three antibacterial agents, two physical fields (pulsed magnetic and electric), and varying frequencies (0.7, 6, and 20 Hz) for durations of 20 and 60 min. As a high-throughput preliminary screen, this work aimed to map the qualitative landscape of bio-electromagnetic interactions. Contrary to the hypothesis of enhanced membrane permeability, ELF PEMW functioned as a biophysical antagonist. The electromagnetic field appeared to trigger membrane hyperpolarization, increasing transmembrane potential and restricting porin-mediated transport of the antibiotic. Simultaneously, the field reduced the zeta-potential of the chitosan nanoparticles, leading to significant colloidal aggregation. These large aggregates were physically excluded from bacterial entry routes, resulting in increased Minimum Inhibitory Concentrations (MICs). Notably, the degree of antagonism was species-specific, suggesting that membrane capacitance and porin density dictate electromagnetic susceptibility. This study reveals a critical bio-electromagnetic trade-off: while physical fields can modulate cellular behavior, poorly tuned parameters can inadvertently fortify bacterial defenses and reduce drug bioavailability. These findings provide a vital “negative roadmap” for future research, highlighting the need for direct electrophysiological mapping of efflux pumps and membrane potentials. This work serves as a foundational step toward precision-targeted, physics-assisted antimicrobial therapies.

**Supplementary Information:**

The online version contains supplementary material available at 10.1038/s41598-026-63235-2.

## Introduction

The rise of antimicrobial resistance necessitates innovative strategies that reduce reliance on traditional high-dosage antibiotic therapies^1,2^. One of the most promising frontiers involves the application of extremely low-frequency pulsed electromagnetic waves (ELF PEMW), which operate at frequencies below 300 Hz and interact with biological systems through non-thermal mechanisms^3,4^. Unlike continuous fields, the rapid on-off switching of ELF PEMW generates transient “shocks” to the bacterial cell membrane, effectively altering membrane potential, surface charge, and permeability^5^. Because biological tissues are non-magnetic and conductive, low-frequency magnetic fields achieve superior penetration compared to electric fields, which attenuate rapidly as a result of tissue conductivity^5^. At the cellular level, these fields exert their influence through the Lorentz force, which modifies ionic trajectories across membranes, and magnetic torque, which can reorient anisotropic membrane phospholipids to alter fluidity^6,7^. Furthermore, resonance phenomena such as Ion Cyclotron Resonance (ICR) and the radical pair mechanism can modulate cellular signaling and increase the production of reactive oxygen species (ROS), thereby shifting the bacterial redox balance^8^.

In the expanding landscape of advanced nanomedicine, a diverse array of engineered vehicles has emerged to address critical therapeutic gaps and combat the global crisis of multidrug resistance (MDR)^9^. Recent studies demonstrate that nanoemulsions, silver nanoparticles, metal oxides, and polymeric carriers such as biocompatible chitosan exhibit significant antibacterial and antibiofilm activities against high-severity ESKAPE pathogens, including *Pseudomonas aeruginosa* and *Staphylococcus aureus*^10^. These innovative platforms bypass traditional resistance pathways through multi-targeted, lethal mechanisms, including direct mechanical membrane disruption, localized oxidative stress via ROS generation, and enhanced intracellular drug penetration^11^. While these individual nanotechnology-based strategies offer powerful standalone capabilities against defensive bacterial matrices, their therapeutic potential can be further maximized^12^. Synergistically pairing advanced polymeric nanocarriers with non-invasive external stimuli represents a promising frontier in modern nanomedicine^13^. By utilizing physical fields to actively modulate biological barriers, this combined approach enhances cellular uptake and maximizes drug delivery, offering a highly effective, synergistic strategy to overcome standard antimicrobial resistance^14^.

This physical intervention creates a synergistic gateway for Chitosan nanoparticles. Chitosan is a deacetylated polymer whose positive amino groups electrostatically bind to and destabilize negatively charged bacterial membranes^15^. When used as a nanocarrier, chitosan protects antibiotics like ciprofloxacin (Cipro) a fluoroquinolone that targets DNA gyrase from degradation while enhancing its targeted delivery^16,17^. Recent studies indicate that ELF PEMW exposure significantly promotes the uptake of these nanoparticle-based drug carriers by inducing magnetoporation. This is a state of enhanced permeability achieved at lower energy thresholds than traditional electroporation^3,18^. This research focuses on clinically significant pathogens, specifically the Gram-negative bacteria *Escherichia coli* and *Pseudomonas aeruginosa*, as well as the Gram-positive bacterium *Staphylococcus aureus*. These species present major therapeutic challenges; *E. coli* frequently modifies its porin channels to limit drug entry, while *P. aeruginosa* utilizes dense biofilms and active efflux pumps to expel antimicrobial agents^19,20^. By combining the mechanical “shock” of ELF PEMW with the membrane-disrupting capabilities of chitosan-loaded ciprofloxacin nanoparticles, this study aims to identify optimal conditions to maximize antibiotic effectiveness at reduced dosages, thereby advancing sustainable antimicrobial practices aligned with global health goals^2,21^.

This study aims to investigate the combined antibacterial effects of chitosan nanoparticles loaded with Cipro and ELF PEMW exposure. Specifically, it focuses on synthesizing Cipro-loaded chitosan nanoparticles with high encapsulation efficiency, applying controlled magnetic and electric exposure at different ELF frequencies (below 20 Hz), and systematically evaluating their antibacterial efficacy against Gram-negative bacteria *Escherichia coli* and *Pseudomonas aeruginosa* and Gram-positive bacteria *Staphylococcus aureus*. The main objectives are to determine whether ELF PEMW exposure enhances the antibacterial activity of Cipro- loaded chitosan nanoparticles by increasing bacterial membrane permeability and drug uptake. Additionally, the study aims to evaluate frequency-dependent effects of ELF PEMW on bacterial viability and biofilm formation. It also seeks to compare the responses of different Gram-negative bacterial strains (*E. coli* and *P. aeruginosa*) to identify variability in susceptibility and how to optimize conditions for maximal antimicrobial synergy.

## Materials and experimental methodology

### Materials

Ciprofloxacin HCl was a kind gift from Pharco Pharmaceutical Industries, Egypt. Medium molecular weight chitosan (Mw = 200 kDa, degree of acetylation > 90%) was purchased from Carl Roth GmbH (Karlsruhe, Germany). Sodium tripolyphosphate (TPP) was purchased from Loba Chemie (Mumbai, India). Glacial acetic acid and diethyl ether were purchased from SD Fine Chemicals (Mumbai, India). Tween 80 was obtained from El-Nasr Pharmaceutical Chemicals (Qaliubiya, Egypt). All other reagents and chemicals were of analytical grade.

### Preparation and characterization of nanoparticles

#### Preparation of chitosan nanoparticles and ciprofloxacin loaded

Chitosan nanoparticles (C-NPs) were synthesized utilizing the ionic gelation approach. This method relies on the electrostatic interaction between the cationic chitosan polymer and anionic tripolyphosphate (TPP) molecules. To prepare the chitosan solution (0.2% w/v), a precisely measured quantity of chitosan was dissolved in a 1% v/v solution of glacial acetic acid while continuously stirred using a magnetic stirrer (Daihan Scientific Co., Seoul, Korea). Following complete dissolution, the pH of the solution was adjusted to 4.5 using 5 N sodium hydroxide (NaOH) and monitored with a pH meter. The solution was subsequently filtered through Whatman filter papers (Whatman Co., London, UK) to remove any undissolved residues. The TPP solution was prepared by dissolving an accurately weighed TPP in distilled water under magnetic stirring^22^. After complete dissolution, the solution was filtered and utilized directly without any modification of pH. C-NPs were synthesized by a single-step addition of 4 mL of TPP solution (0.15% w/v) into 10 mL of chitosan solution, while maintaining a stirring speed of 1200 rpm. The mixture was stirred continuously for 30 min at ambient temperature to facilitate the formation of a uniform and stable colloidal system. Ciprofloxacin HCl, a broad-spectrum antibiotic, is incorporated during C-NPs formation process to prepare ciprofloxacin HCl-loaded chitosan nanoparticles (Cipro-C-NPs). The drug is dissolved (0.05%w/v) in the chitosan solution before adding TPP to allow efficient entrapment^23^.

#### Physicochemical characterization

##### Determination of entrapment efficiency (%EE)

To determine the %EE of Cipro-C-NPs, 1 ml sample was placed inside a dialysis bag (VISKING dialysis tube, Mwt cutoff = 12,000 Da, SERVA, electrophoresis, Germany), then the bags were placed in centrifuge tubes containing distilled water (10 ml) and was centrifuged at 4000 rpm for 30 min (Centrifuge, Smic, Shanghai, China)^24^. The free unentrapped drug diffuses through the dialysis membrane and was quantified using UV-visible spectrophotometer at 278 nm (UV-1800 Shimadzu Spectrophotometer, Kyoto, Japan). Placebo C-NPs were used to assess the contribution of NPs components to UV absorption. The %EE was determined using the following equation^25^:

$$\:\mathrm{\%}Entrapment\:efficiency\:\left(\%EE\right)=\:\frac{Total\:drug\:-\:Unentrapped\:drug}{Total\:drug\:}\times\:100$$

##### Particle size, polydispersity index, and zeta potential

The particle size (PS), Polydispersity index (PDI) and zeta potential (ZP) of C-NPs and Cipro-C-NPs were determined by dynamic light scattering technique (DLS) using Zetasizer Nano ZS (Malvern, Instruments Ltd., Malvern, UK). Measurements were conducted at 25 °C^26^. Each sample was measured in triplicates and results were represented as mean value ± SD.

##### Fourier-transform infrared spectroscopy (FT-IR)

FTIR spectra were acquired for chitosan, TPP, C-NPs, Ciprofloxacin HCl and Cipro-C-NPs. Each specimen was subjected to FTIR spectroscopic analysis using an Agilent FTIR spectrophotometer (Agilent, USA), scanning across the wavenumber range from 4000 to 500 cm^−1^^27^.

##### Structure elucidation and morphological characterization

Transmission electron microscopy (TEM, Model JEOL-JSM-1400 PLUS, Tokyo, Japan) was used for detailed structure examination of Cipro-C-NPs. Samples were dropped onto a carbon-coated copper grid. Then, they were left for 1 min to allow vesicles adherence to the carbon substrate. The excess dispersion was removed with a filter paper. Negative staining using 2% uranyl acetate solution (w/w) was directly made on the deposit for 45s. Then, the air-dried samples were directly examined under the TEM^28^.

### Methodology of electromagnetic exposure

All exposure protocols adhere strictly to the safety guidelines established by the International Commission on Non-Ionizing Radiation Protection (ICNIRP) and the World Health Organization (WHO)^29^. This study expands upon existing research regarding the intersection of nanotechnology and electromagnetic wave (EMW) exposure. Specifically, we are further investigating this field by utilizing optimized parameters such as frequency range, field intensity, and duration derived from our previous findings.

#### Magnetic field exposure system

The experimental framework utilizes a custom-engineered, extremely low-frequency (ELF) magnetic field system designed around a precision solenoid. The solenoid is constructed with 1020 turns of high-conductivity copper wire, featuring a DC resistance of 11.2 Ω and a longitudinal core length of 8.5 cm. To create an “interrupted” field characteristic, the solenoid is integrated into an electronic switching circuit driven by a 65 mA constant current power supply^30^. This configuration produces a 50% duty cycle square-wave modulation, alternating between a peak magnetic flux density (B) and a zero-field state. Biological specimens are positioned coaxially at a distance of 0.5 cm from the solenoid’s distal face, where the flux density is verified at 18 mT ± 2% using a Bell Model 4048 Gauss/Tesla meter. Experimental variables are strictly controlled, with field kinetics modulated across various exposure durations and frequency intervals to ensure compliance with ICNIRP safety thresholds and longitudinal laboratory data^31^.

#### Electric field exposure system

The experimental environment was established using a parallel-plate capacitor apparatus, consisting of two 4 cm diameter circular brass electrodes separated by a fixed 5 cm gap. This system was specifically configured for interrupted electric field (EF) protocols, utilizing a high-voltage pulse generator to modulate a 6 V DC input into a 50% duty cycle square-wave. Under these conditions, an applied voltage of 250 V generated a verified field intensity of 5.11 ± 0.13 kV/m at the sample locus, with the waveform precisely monitored via a GOS-620 oscilloscope. To ensure field homogeneity and experimental integrity, bacterial supernatants in borosilicate tubes were positioned on a dielectric platform at the setup’s geometric center^32^. The procedure was conducted at a strictly governed ambient temperature of 27 °C across various exposure durations and frequency intervals. To maintain a uniform suspension and prevent bacterial sedimentation, periodic manual agitation was performed throughout the duration of the treatment.

**Fig. 1:**
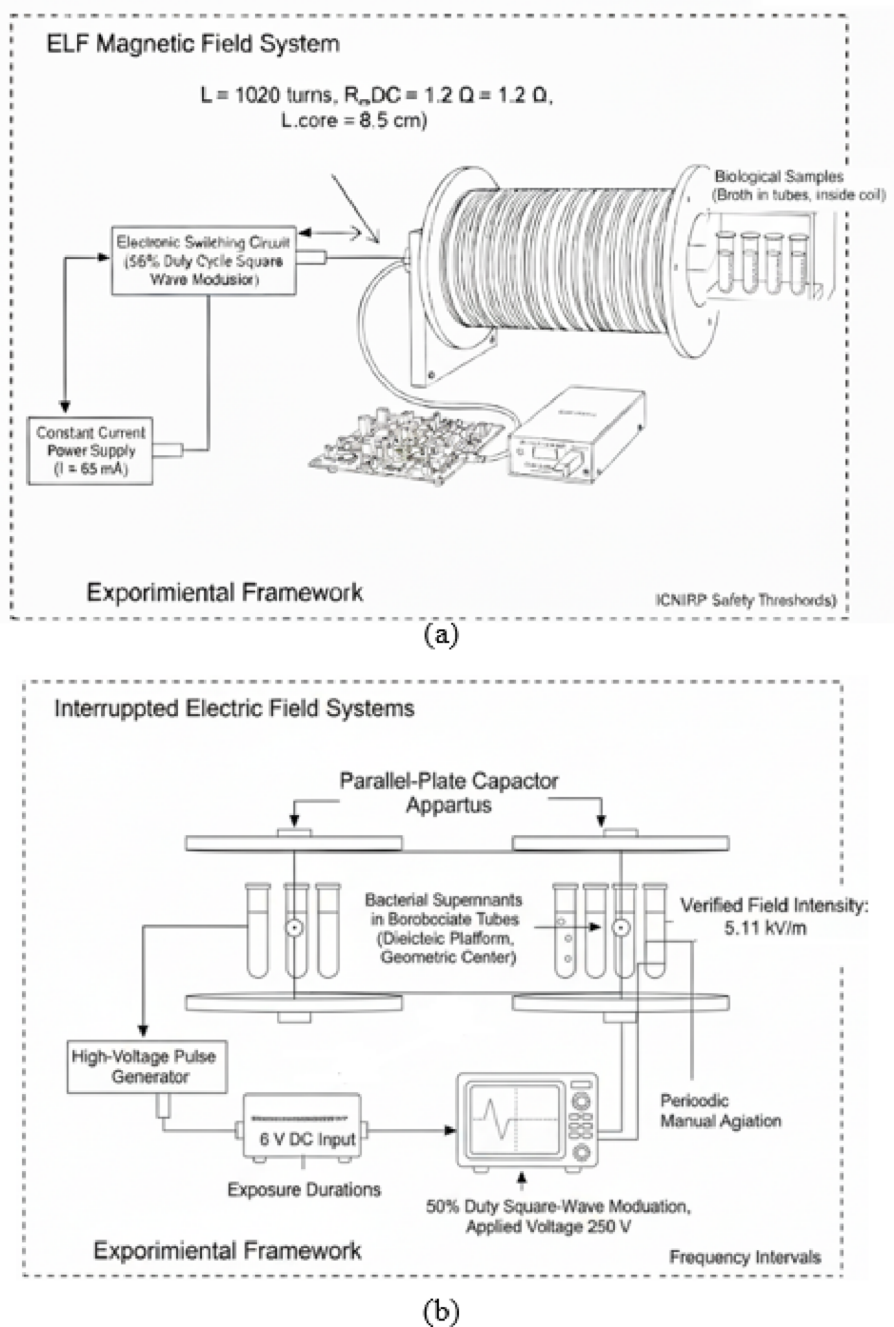
(**a**) Schematic for the exposure PMF system. (**b**) Schematic for the exposure PMF system PEF system.

### Antibacterial efficacy and biofilm dynamics

#### Bacterial strains and ethical considerations

The bacterial isolates utilized in this study were obtained as anonymized, secondary cultured isolates from routine diagnostic laboratory collections in Alexandria, Egypt. The authors had no direct involvement with human participants, sample collection procedures, or access to patient identifiers, medical records, or demographic data at any stage of the study. The isolates were supplied exclusively as pre-existing bacterial cultures for research purposes; accordingly, the study did not constitute human subject research and was exempt from ethical approval and informed consent requirements in accordance with institutional and international research ethics guidelines. All experiments were conducted following standard laboratory biosafety guidelines.

The study specifically utilized *Staphylococcus aureus* (Gram-positive), *Escherichia coli*, and *Pseudomonas aeruginosa* (Gram-negative)^33^. Species specific confirmation and viability check of these pre-existing cultures were performed using selective and differential media. This included MacConkey agar to evaluate lactose fermentation characteristics for *E. coli*, Pseudomonas agar to verify the characteristic pyocyanin production of *P. aeruginosa*, and Mannitol Salt Agar for *S. aureus*^34^. Antibacterial activity was evaluated using Cipro (stock solution 400 mg/100 ml), Chitosan nanoparticles (20 mg/10 ml), and Chitosan loaded with Cipro nanoparticles (5 mg/10 ml) against these three isolates.

#### Antibiotic susceptibility testing

Antibiotic susceptibility was assessed using the Kirby-Bauer disk diffusion method^35^, a standardized technique in which antibiotic-impregnated paper disks are placed on an agar plate spread with the test organism. After incubation, the diameter of the inhibition zone around each disk is measured to determine the bacterial strain’s sensitivity to the antibiotic. A panel of antibiotics including amikacin, ampicillin, amoxicillin/clavulanic acid, cefotaxime, ceftazidime, ciprofloxacin, levofloxacin, and tobramycin was tested. Inhibition zones were measured after incubation at 37 °C. As this study prioritized a qualitative assessment of potential trends, detailed numerical values were not recorded. Instead, visual observations were used to identify notable shifts in susceptibility patterns.

#### Minimum inhibitory concentration determination

MIC values for Cipro, C-NPs, and Cipro-C-NPs were determined using a two-fold serial dilution method in sterile 96-well microtiter plates. Wells were inoculated with bacterial suspensions adjusted to 10^6^ CFU/mL and incubated at 35–37 °C for 14 h. MIC was recorded as the lowest concentration showing no visible growth. To evaluate the efficacy of the combined treatment relative to each individual component, the Fractional Inhibitory Concentration Index (FICI) was calculated as follows^35–38^:

$$\:\mathrm{FICI}=\:\varSigma\:\:\frac{\mathrm{MIC\:of\:}\mathrm{treatment\:}\mathrm{in\:combination}}{\mathrm{MIC\:of\:}\mathrm{treatment\:}\mathrm{alone}}$$

$$\begin{array}{ccccc}& Results\:were\:interpreted\:as\:synergistic\:if\:\sum {FICI \le \:\:0.5} \:,\\& \quad additive\:if0.5 < \sum {FICI \le \:1.0} ,indifferent\:if1.0\\& \quad > \sum {FICI \le \:\:4.0} \:,and\:antagonistic\:if\:\sum {FICI > 4.0} \end{array}$$

#### Biofilm assays

##### Biofilm formation capacity

Biofilm formation was evaluated using a modified microtiter plate method^39^. Bacterial sus- pensions adjusted to CFU/mL (colony-forming units per milliliter, representing the number of viable bacterial cells capable of forming colonies) were seeded into flat-bottom 96-well plates and incubated overnight. Wells were washed, air-dried, fixed with ethanol, stained with 1% crystal violet, and eluted with 33% glacial acetic acid. Absorbance at 630 nm was measured to quantify biofilm mass. As in the antibiotic susceptibility test, changes in biofilm mass were primarily assessed through relative, visually observable differences in staining intensity and absorbance.

##### Antibiofilm activity of formulations

The antibiofilm effects of Cipro, C-NPs, and Cipro-C-NPs were tested by treating pre-formed biofilms. After 24 h, wells were washed 3 times with saline, fixed with 200 µl of 99% methanol for 15 min, stained with crystal violet, and absorbance was measured at 630 nm to determine remaining biofilm mass. To clearly illustrate the inhibitory effects on biofilm formation, a representative concentration was selected from each treatment group for comparative analysis^40^. Biofilm reduction was quantified using the formula: $\:1-\left({\mathrm{OD}}_{\mathrm{treated}}/{\mathrm{OD}}_{\mathrm{untreated}}\right)\times\:100$^41^. This approach allows for a streamlined comparison of the synergistic impact of treatment and PEF and PMF exposure across different bacterial strains.

##### Detection of virulence factors

Qualitatively, hemolysin production was evaluated on blood agar plates for *E. coli* and *S. aureus*^42^. In addition, the pyocyanin pigment production as a virulence factor of *P. aeruginosa* was assessed by its intensity on glycerol-supplemented Pseudomonas agar^43^.

### Treatment protocol and Groups

The work included a multi-factorial experimental design to evaluate the antibacterial efficacy of Cipro, C-NPs and Cipro-C-NPs and the synergism of exposure to pulsed magnetic (PMF) and electric fields (PEF) against *E. coli*, *P. aeruginosa* and *S. aureus*^44,45^. For comparison purposes, the bacteria grown with no treatment at all (untreated bacterial strains) were considered as negative control samples, while the bacteria treated by Cipro were considered as positive controls. The synergistic antibacterial efficacy was evaluated for each bacterial strain by supplementation with the antibacterial agents (Cipro, C-NPs, and Cipro-C-NPs), followed by exposure to PMF and PEF^30–32,46,47^. These fields exposures were tested at three frequencies (0.7 Hz, 6 Hz, and 20 Hz) and two durations (20 min and 60 min)^30–32,44–47^. The chosen frequencies and exposure periods were selected based on parameters established in previous research. Similarly, the synergistic antibacterial efficacy was also assessed following exposure to pulsed electric fields (PEF) using the same frequencies and durations. Due to the large number of experimental groups required to explore the multi-factorial synergism of agents and fields, each exposure condition was tested on a single bacterial culture per group. Each bacterial strain is represented by a single clinical isolate (*n* = 1) that was subjected to 7 conditions. Each experiment was done in triplicate. As such, this initial study is qualitative and provides insight into potential trends but is not statistically powered for quantitative comparison.

### Statistical analysis and data representation

To ensure rigorous data transparency given the specialized nature of the PEF and PMF exposures, all experimental outcomes are reported as the mean of three independent trials. Due to the high precision required for electromagnetic synchronization, all data, including fractional indices (FICI) and normalized percentages, are presented with error bars^48^. It represents the uncertainty margin calculated from the total observed variance between trials^49^. These bars represent the calculated mean adjusted for both instrumental precision and biological variance observed during the experimental runs^50^.

## Results

### Synthesis and characterization of cipro-loaded chitosan nanoparticles

**Fig. 2:**
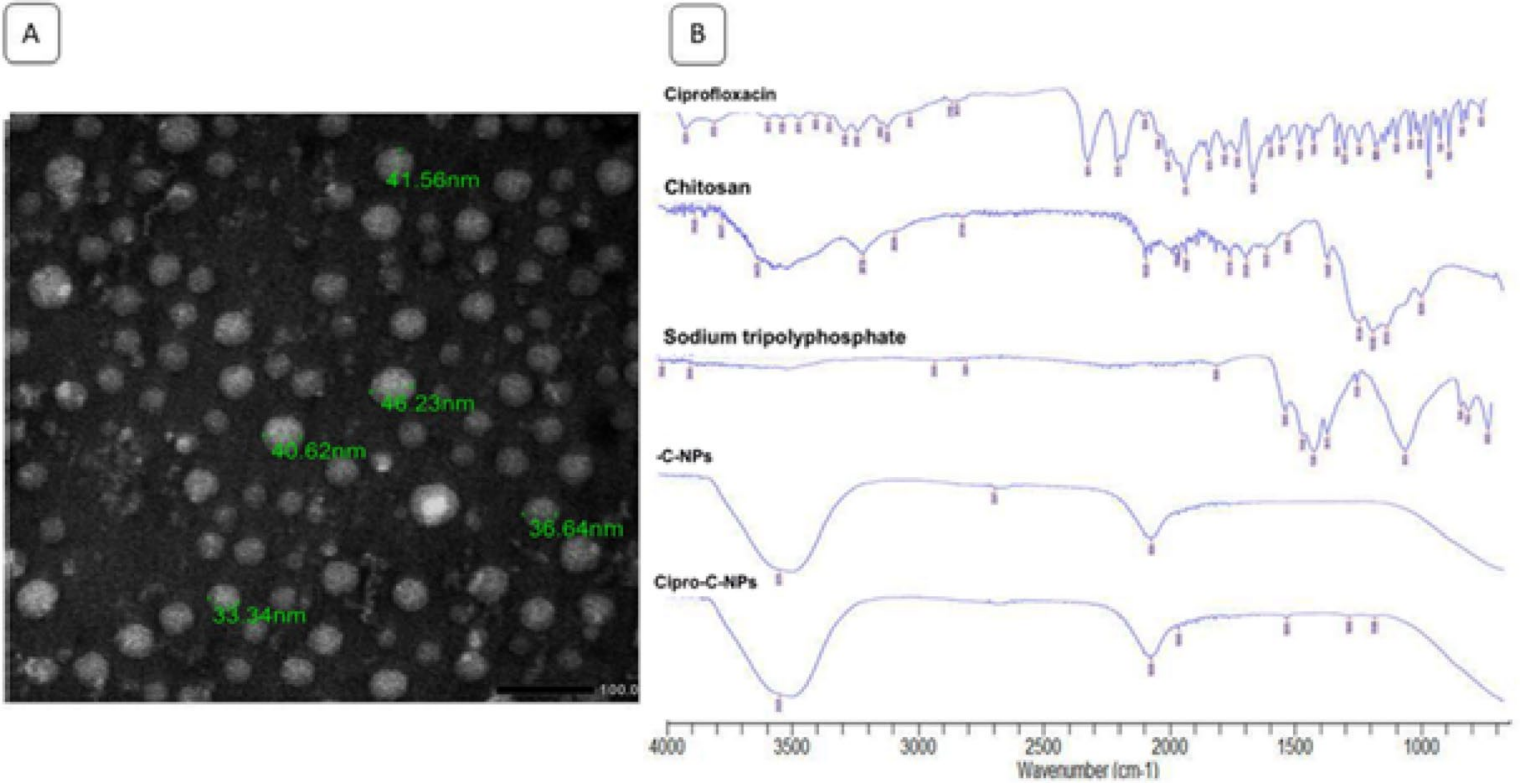
(**A**) TEM micrograph of Cipro-C-NPs, (**B**) Fourier transform infrared spectra of: drug; ciprofloxacin, chitosan, sodium tripolyphosphate, C-NPs and Cipro-C-NPs.

Ciprofloxacin-loaded chitosan nanoparticles (Cipro-C-NPs) were prepared using the previously reported ionic gelation method by the ionic interaction between the cationic amino group in chitosan and the anionic phosphate groups in TPP. As shown in Table 1, the prepared Cipro-C-NPs showed a remarkable increase in PS and PDI (160 ± 2.6 nm and 0.34 ± 0.06) compared to the C-NPs (122 ± 1.2 nm and 0.22 ± 0.04) due to the Cipro loading. Moreover, the low PDI values indicate homogenous dispersions. On the other hand, Cipro-C-NPs showed a decrease in ZP (+ 40 ± 2.51 mV) compared to C-NPs (+ 53 ± 1.32 mV). This decrease might be due to partial chitosan neutralization by the loaded Cipro. Positively charged nanoparticles with ZP above ± 30 mV indicates stable dispersion due to electrostatic repulsion between the charged particles. Regarding the %EE, Cipro-C-NPs showed 97 ± 0.57% of Cipro entrapped into the prepared nanoparticles. This might be due to the ionic interaction between the positively charged chitosan and the COOH group in Cipro. The TEM analysis of Cipro-C-NPs shows spherical nanoparticles with a uniform size distribution (Fig. 2A). The particles are well dispersed with no significant aggregation observed, indicating good colloidal stability. TEM measurements showed slightly smaller particle sizes compared with those obtained by DLS. This difference can be explained by the distinct sample preparation methods used in each technique. For TEM analysis, samples were dehydrated through vacuum drying inside the TEM chamber, whereas DLS measurements were performed on samples dispersed in a hydrated solution^51^. Crucially, our dynamic light scattering (DLS) data serves as the comparative baseline to track structural changes post-loading, where the significant shift from 122 ± 1.2 nm for pure chitosan nanoparticles to 160 ± 2.6 nm for Cipro-C-NPs clearly substantiates the physical payload accommodation. Because free ciprofloxacin is a small molecular drug that dissolves entirely in solution without possessing an independent nanoparticulate structure, capturing the final spherical, non-aggregated morphology via TEM confirms the successful core assembly and uniform encapsulation within the polymer matrix compared to the looser, un-crosslinked native polymer chains.

**Table 1:** Characterization of the prepared formulations.

Formulation code	Characterization			
	PS (nm)	PDI	ZP (mV)	% EE
C-NPs	122 ± 1.2	0.22 ± 0.04	+ 53 ± 1.32	–
Cipro-C-NPs	160 ± 2.6	0.34 ± 0.06	+ 40 ± 2.51	97 ± 0.57

*PS, Particle size; PDI, Polydispersity index; ZP, Zeta potentia; EE, Entrapment efficiency.

### FTIR spectroscopic analysis

FTIR analysis was performed to confirm the interaction of the chitosan, TPP, and Cipro. The obtained FTIR spectra confirm successful chitosan nanoparticles formation and drug loading. Key observations include the interaction of chitosan with TPP (ionic gelation) and the encapsulation of Cipro via hydrogen bonding and electrostatic interactions. The FTIR spectra of chitosan, TPP, C-NPs, Cipro HCl and Cipro-C-NPs. are presented in Fig. 2B.

Chitosan shows characteristic FTIR peaks corresponding to its functional groups. The broad absorption bands around 3400–3200 cm^−1^ due to intermolecular and intramolecular hydrogen bonding of O–H and N–H stretching. C–H stretching bands are observed at 2900–2850 cm^−1^. Amide I (C=O stretching) shows broad bands at around 1650 cm^−1^, due to residual acetyl groups and amide II (N–H bending) shows a characteristic peak at 1550 cm^−1^ due to amine groups. C–O–C stretching is observed at 1150–1020 cm^−1^ which is related to the glycosidic linkages in chitosan. TPP exhibits P=O stretching peak around 1210–1250 cm^−1^ and P–O stretching peaks in the range of 900–1150 cm^−1^, indicating phosphate groups. Upon nanoparticles formation with TPP in C-NPs and Cipro-C-NPs, changes in chitosan’s FTIR spectrum confirm the crosslinking. The amide II band at 1550 cm^−1^ shifts or reduces, indicating interaction between chitosan and TPP. Bands at 1150–900 cm^−1^ appeared, confirming P–O–C bonding between chitosan and TPP. Reduced intensity of O–H/N–H stretching suggests hydrogen bonding and nanoparticles formation. This result indicated that the NH_3_^+^ groups of chitosan were crosslinked with the TPP groups of sodium polyphosphate, which helped to enhance both the inter- and intra-molecular interactions within the chitosan nanoparticles^27^.

Ciprofloxacin HCl has characteristic peaks at around 3440 cm^−1^ for O–H stretching (hydroxyl group). C=O stretching band is observed at 1700 cm^−1^. C–F stretching shows a strong absorption band at 1250 cm^−1^, indicating the presence of fluorine. C–N stretching is observed at around 1300 cm^−1^ due to the quinolone ring. In the case of Cipro-C-NPs, the peak at 3421 cm^− 1^ corresponding to the hydroxyl groups in chitosan was shifted to 3428 cm^− 1^, indicating that there could be some hydrogen bonding between the Cipro and chitosan nanoparticles. The bands at 1657 and 1595 cm^− 1^ (amide I and amide II band), as clearly observed in chitosan, were also decreased in Cipro-C-NPs, while two new absorption bands appeared at 1633 and 1536 cm^− 1^ in Cipro-C-NPs. The band at 1299–1155 cm^− 1^ was related to the C–F band stretching of Cipro^27^.

### Baseline antimicrobial activity and chemical synergy

**Fig. 3:**
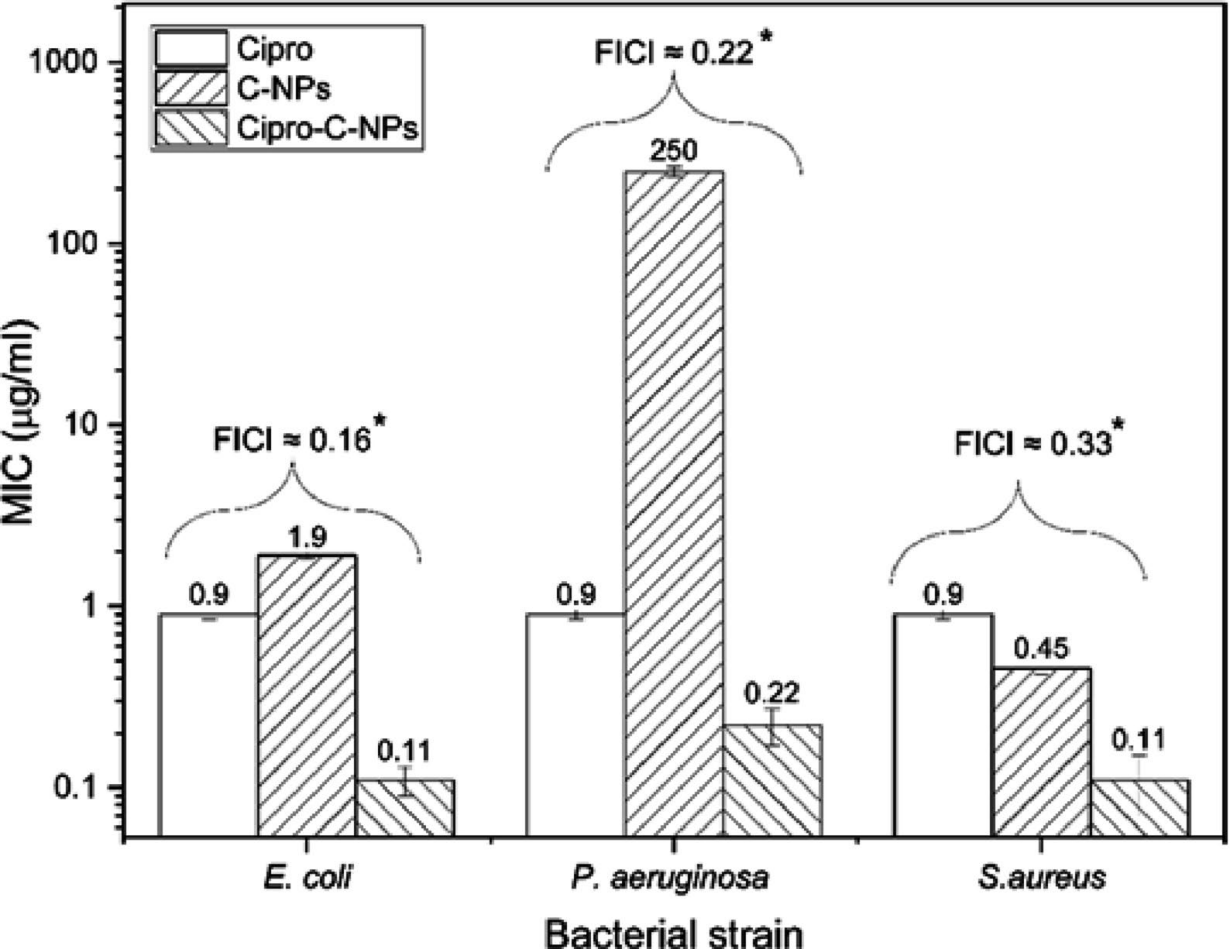
The MIC of Cipro, C-NPs, and their combination Cipro-C-NPs against *E. coli*, *P. aeruginosa*, and *S. aureus*. The Y-axis is presented on a logarithmic scale (µ/ml). FICI Scales: *≤ 0.5: Synergy.

Essentially, the antimicrobial efficacy of Cipro, C-NPs, and Cipro-C-NPs, both independently and in combination with PEF and PMF exposures were evaluated. The treatments were tested against the two primary classifications of bacterial pathogens Gram-positive and Gram-negative bacteria. Gram-negative organisms are needed to avoid concluding a single, non-generalizable data point; one Gram-positive is sufficient to provide the contrast. Our primary objective was to elucidate how applied treatments and field exposures would affect the susceptibility of organisms under test. Our analysis begins with determining the MIC, which serves as the essential starting point for evaluating how bacteria respond to Cipro, C-NPs, and Cipro-C-NPs. The Gram-positive *S. aureus*, the Gram-negative *E. coli*, and *P. aeruginosa* were chosen as pathogenic candidates implicated in majority of community acquired diseases. Initial identification confirmed the classic phenotypes for all isolates: *E. coli* (pink, mucoid on MacConkey agar), *P. aeruginosa* (bluish-green on Pseudomonas agar), and *S. aureus* (golden-yellow on MSA). By identifying the lowest dose that stops visible bacterial growth, we establish a precise baseline that is necessary for all further testing. This measurement ensures that our subsequent evaluations are grounded in the exact sensitivity of the specific strain being studied. As shown in Fig. 3, the Cipro-C-NPs combination exhibited significantly higher antibacterial potency compared to the individual components across all tested strains. The MIC for Cipro was found to be ≥ 0.9 µg/ml for all bacterial strains tested.

The influence of C-NPs alone showed a slightly higher MIC value for *E. coli* be ≥ 1.9 µg/ml, a slightly lower value was observed for *S. aureus* be ≥ 0.45 µg/ml, and a dramatic increase for *P. aeruginosa*, be ≥ 250 µg/ml. Remarkably, while C-NPs alone showed a high MIC against *P. aeruginosa* (~ 250 µg/ml), the combination with Cipro resulted in a five-fold reduction of the antibiotic’s MIC, dropping from nearly 1.0 µg/ml to approximately 0.2 µg/ml. To evaluate the efficacy of the combined treatment relative to each individual component, the Fractional Inhibitory Concentration Index (FICI) was calculated as aforementioned in the materials and methods section and mentioned in the Fig. 3 (* ≤ 0.5: Synergy). The calculated FICI values for all three strains were ≤ 0.5 (ranging from 0.16 to 0.33), indicating a strong synergistic effect. This suggests that the nanoparticles significantly enhance the efficacy of Cipro, potentially bypassing existing resistance mechanisms in both Gram-positive and Gram-negative models.

### Antimicrobial potentiation by combined field exposures (PEF/PMF) and Cipro-C-NPs

Having established the baseline chemical synergism between Cipro and chitosan nanoparticles in the absence of physical stimuli, we evaluated how pulsed electric fields (PEF) and pulsed magnetic fields (PMF) further potentiate these antimicrobial effects. Because the cellular response to both the nanoparticles and the applied fields is inherently species-specific due to fundamental differences in cell wall architecture (Gram-positive vs. Gram-negative), the subsequent results are organized systematically by biological target. This approach facilitates the identification of the optimal frequency and duration parameters required for maximal therapeutic enhancement. Internal variance between experimental trials remained consistently low across these complex setups.

#### Effects on *Escherichia coli*

**Fig. 4:**
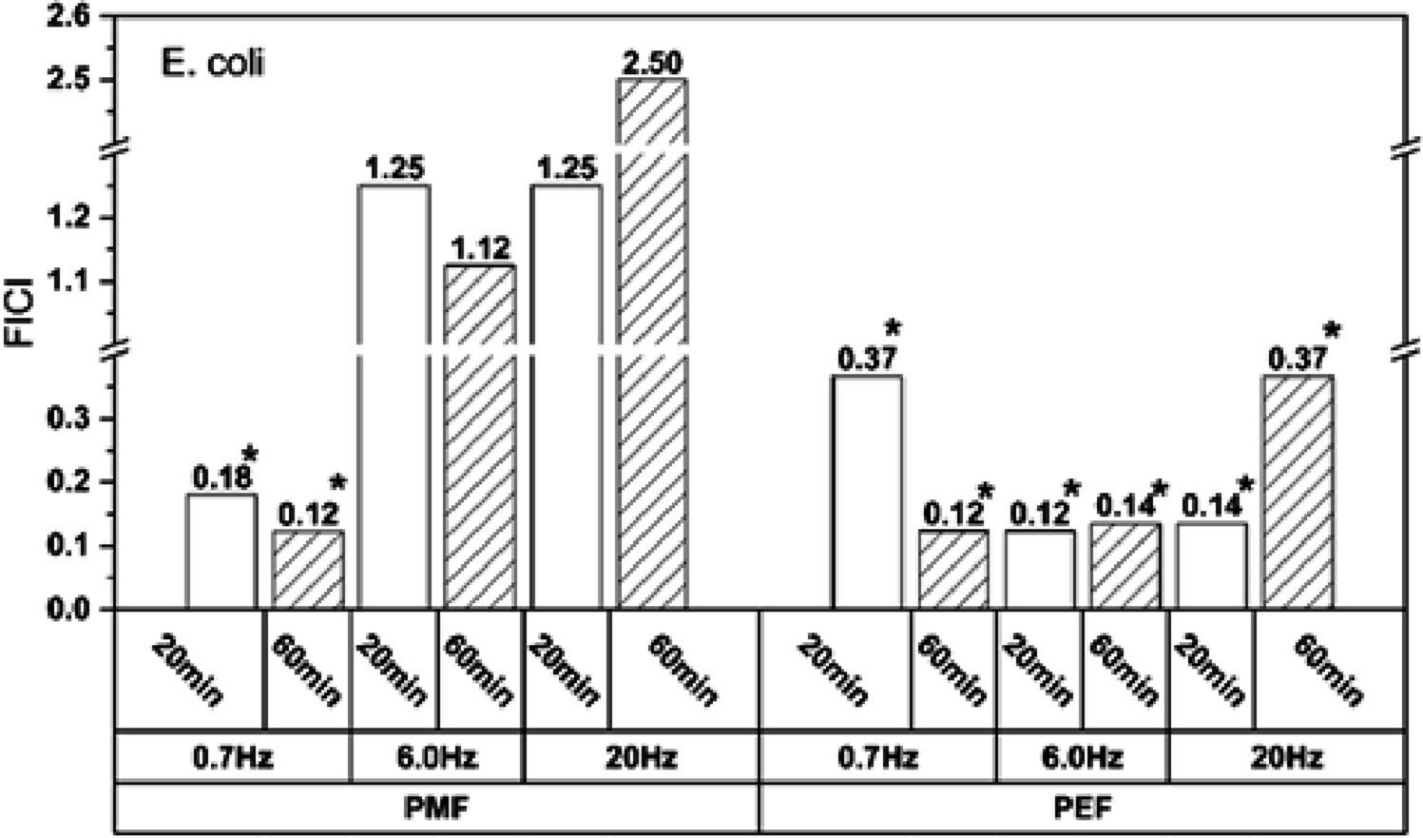
The efficacy FICI of the combined exposure relative to each individual treatment of Cipro, C-NPs, and their combination Cipro-C-NPs against *E. coli*. FICI Scales: * ≤ 0.5: Synergy.

**Fig. 5:**
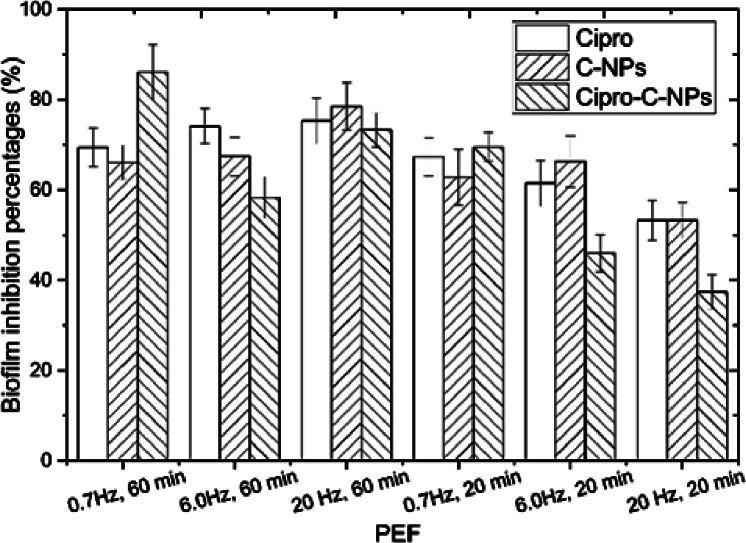
The comparative biofilm inhibition percentages represent the synergistic impact of exposure to PEF at different frequencies, periods against *E. coli*.

**Fig. 6:**
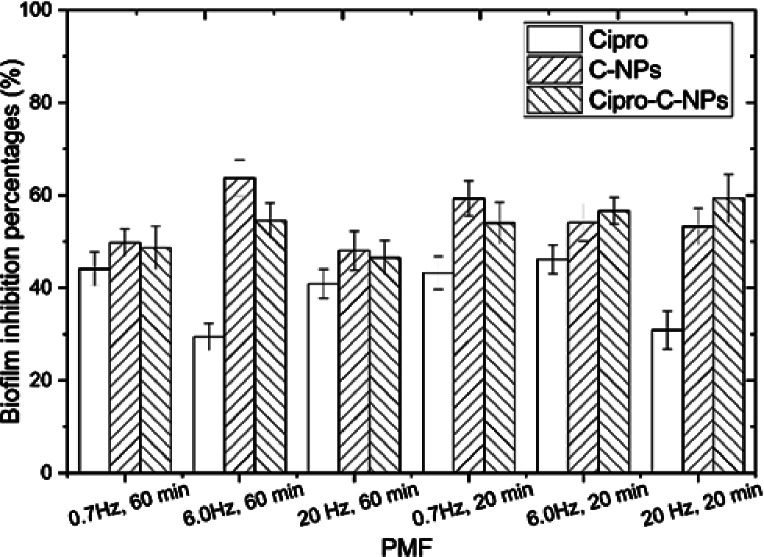
the comparative biofilm inhibition percentages represent the synergistic impact of exposure to PMF at different frequencies, periods against *E. coli*.

The combined application of physical fields and Cipro-C-NPs against *E. coli* demonstrated divergent outcomes highly dependent on the exposure type:

- MIC endpoints and synergism: *E. coli* exhibited a broad, robust synergistic response (FICI ≤ 0.5) to PEF across all tested frequencies (0.7, 6, and 20 Hz) and exposure times (20 and 60 min) (Fig. 4). Conversely, synergy with PMF was highly selective, occurring exclusively at 0.7 Hz for both exposure durations. Under all other PMF conditions, the interaction was merely indifferent (1.0 < FICI ≤ 4.0), meaning the magnetic field did not further reduce the MIC beyond the baseline efficacy of Cipro-C-NPs alone.
- Antibiogram screening: Exposure to PEF particularly at 0.7 Hz for 60 min significantly enhanced antibiotic sensitivity, successfully converting resistant profiles into sensitive ones for multiple agents, most notably Colistin (CT), Tetracycline (TE), and Cefuroxime (CXM). Higher PEF frequencies (6 and 20 Hz) showed more variable responses. In stark contrast, PMF exposure led to a consistent reduction in inhibition zones, with several antibiotics (Aztreonam (ATM), Ofloxacin (OFX), and Ceftriaxone (CRO)) reaching complete resistance regardless of frequency or duration.
- Virulence profile: Combined exposure to both PEF and PMF failed to induce any phenotypic changes in hemolysin production; *E. coli* remained consistently non-hemolytic across all experimental parameters.
- Biofilm inhibition: Biofilm reduction exhibited an exposure type and time dependent response (Figs. 5 and 6). The biofilm was most susceptible to PEF at 0.7 Hz for 60 min, reaching a maximum inhibition of 86% (with a minimum effect of 37% at 20 Hz for 20 min). On the other hand, the PMF treatment groups demonstrated a remarkably narrow, stable, and uniform inhibitory range, peaking at 59% (20 Hz, 20 min) with a minimum baseline of 47% (20 Hz, 60 min).

#### Effects on *Pseudomonas aeruginosa*

**Fig. 7:**
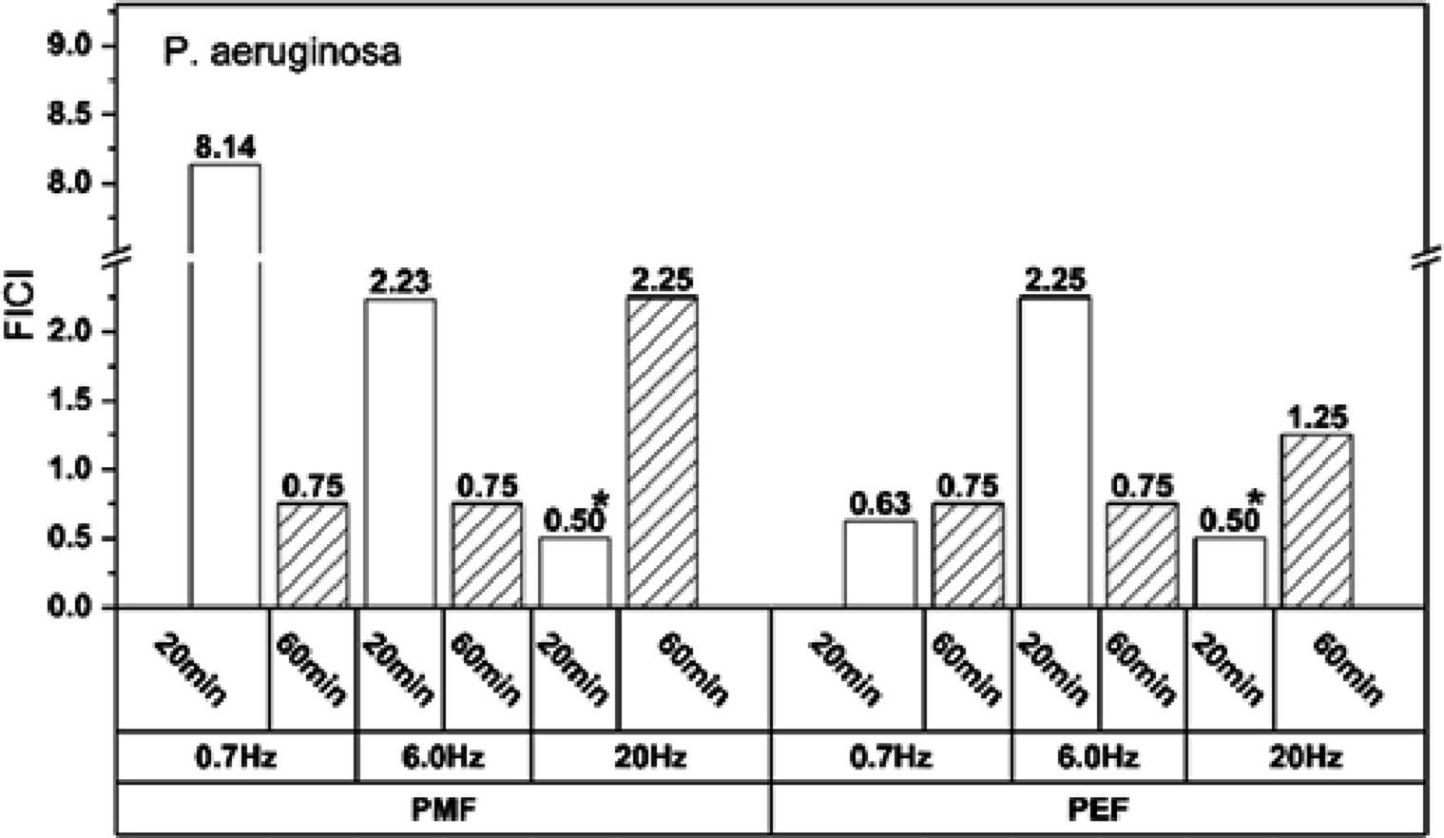
the efficacy FICI of the combined exposure relative to each individual treatment of Cipro, C-NPs, and their combination Cipro-C-NPs against *P. aeruginosa*. FICI Scales: * ≤ 0.5: Synergy.

**Fig. 8:**
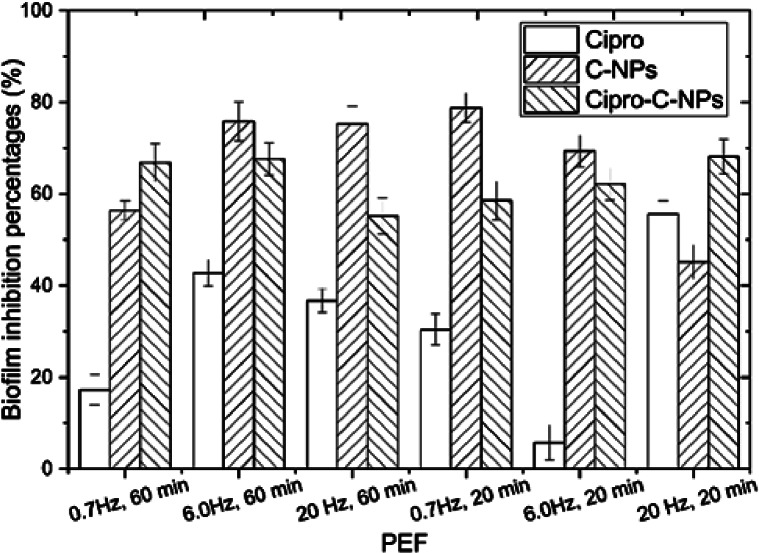
the comparative biofilm inhibition percentages represent the synergistic impact of exposure to PEF at different frequencies, periods against *P. aeruginosa*.

**Fig. 9:**
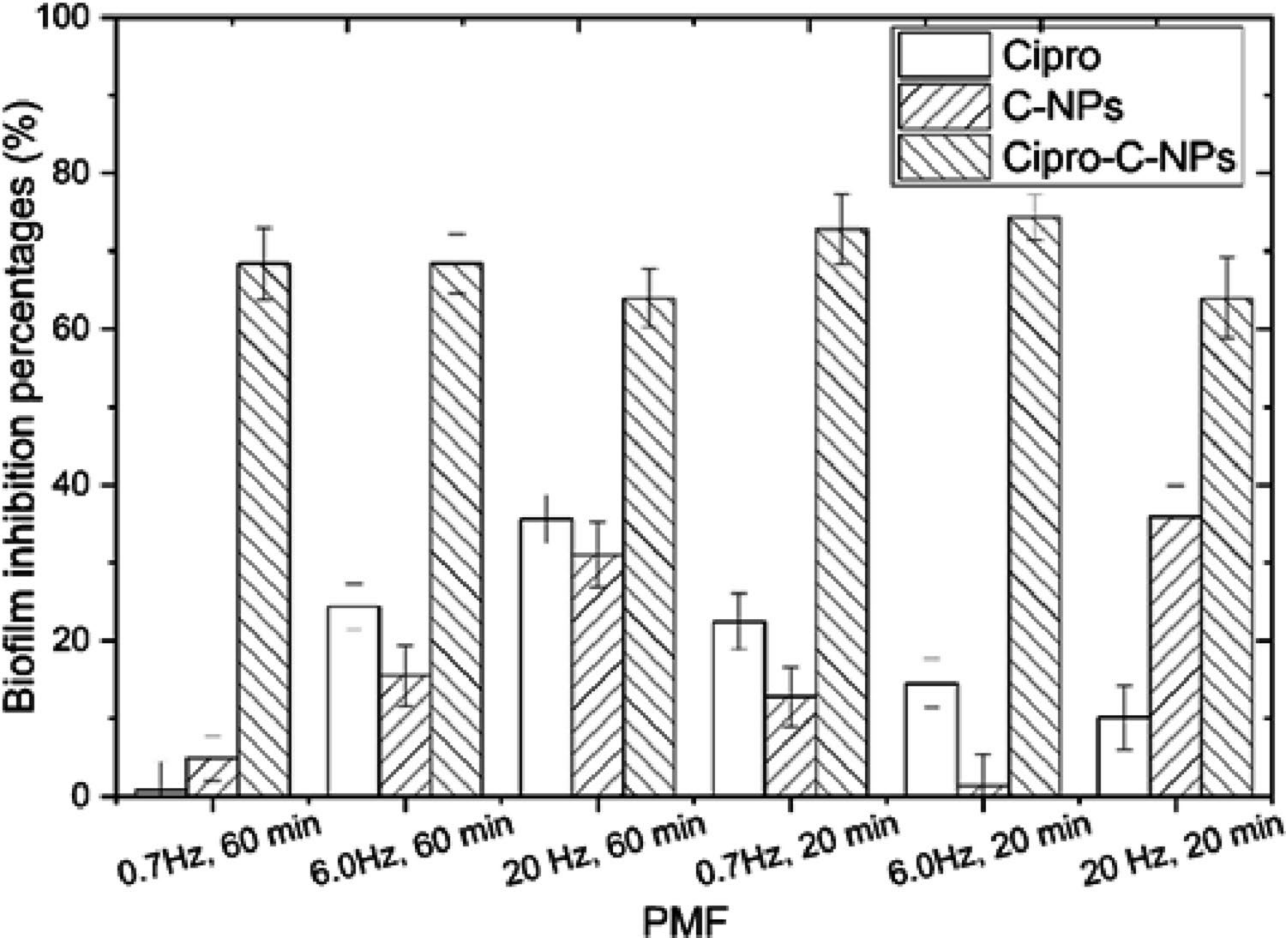
the comparative biofilm inhibition percentages represent the synergistic impact of exposure to PMF at different frequencies, periods against *P. aeruginosa*.

Reflecting its high adaptive threshold, *P. aeruginosa* displayed highly specific, parameter-dependent responses to the electromagnetic treatments:

- MIC endpoints and synergism: Clear synergistic enhancement (FICI ≤ 0.5) was narrow, observed specifically when *P. aeruginosa* was exposed to 20 Hz for 20 min under *both* PEF and PMF conditions (Fig. 7). Other combinations resulted in additive or indifferent responses (0.5 < FICI ≤ 4.0). Notably, PMF exposure at 0.7 Hz for 20 min resulted in true antagonism (FICI > 4.0), where the field treatment actively interfered with the nanoparticle action.
- Antibiogram screening: Under PEF exposure, the isolate generally shifted toward increased antibiotic resistance, reducing zone diameters for most agents. However, critical exceptions occurred where PEF significantly increased sensitivity to CT, Azithromycin (AZM), and ATM. PMF exposure resulted in only marginal fluctuations, suggesting its inductive effects were insufficient to perturb the robust defenses of this species.
- Virulence profile (pyocyanin): Production of the redox-active virulence factor pyocyanin showed highly divergent trends between the fields. PEF exposure at 20 Hz for 20 min significantly enhanced pyocyanin intensity. Conversely, high-frequency, longer-duration PMF exposure (20 and 60 min) completely abolished pyocyanin production.
- Biofilm inhibition: Biofilm dynamics remained within a relatively narrow, stable range for both field types, demonstrating a highly reliable biological response (Figs. 8 and 9). Maximum inhibition reached 68% for PEF (0.7 Hz, 60 min) and 74% for PMF (20 Hz, 20 min); minimum inhibition values were 55% (PEF: 0.7 Hz, 20 min) and 64% (PMF: 0.7 Hz, 60 min).

#### Effects on *Staphylococcus aureus*

**Fig. 10:**
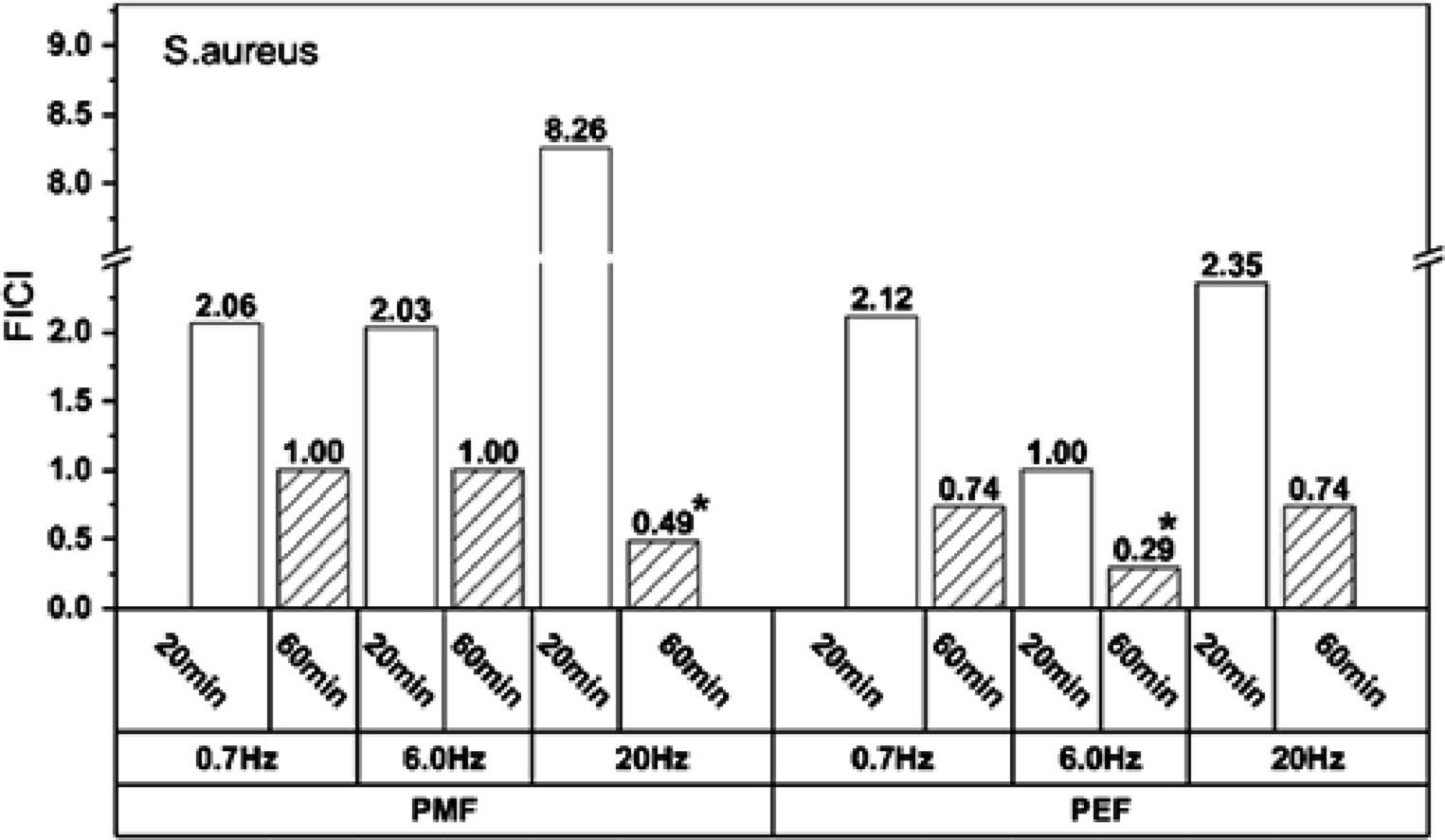
the efficacy FICI of the combined exposure relative to each individual treatment of Cipro, C-NPs, and their combination Cipro-C-NPs against *S. aureus*. FICI Scales: * ≤ 0.5: Synergy.

**Fig. 11:**
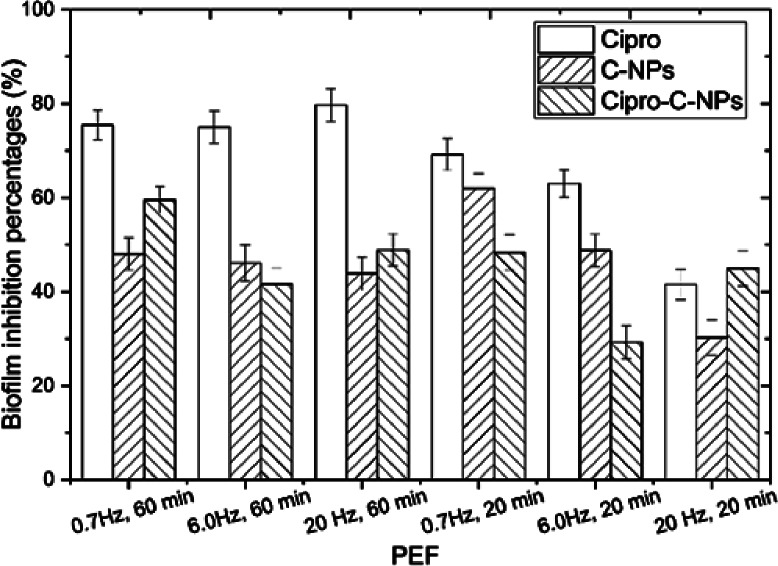
the comparative biofilm inhibition percentages represent the synergistic impact of exposure to PEF at different frequencies, periods against *S. aureus*.

**Fig. 12:**
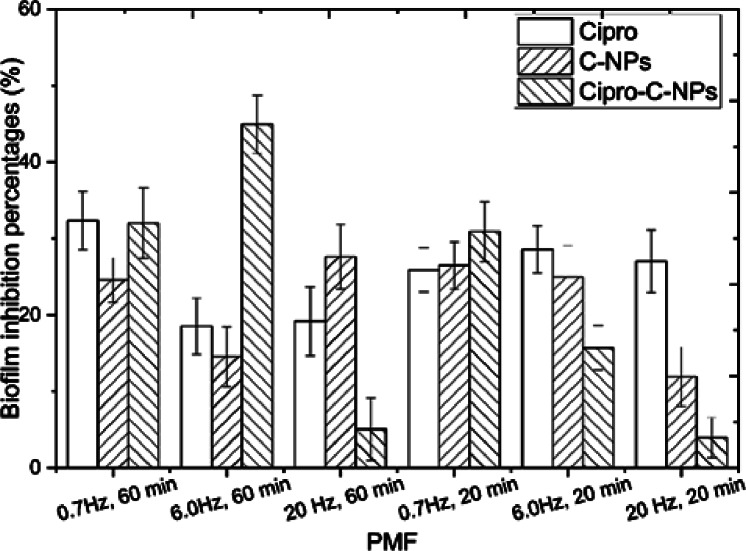
the comparative biofilm inhibition percentages represent the synergistic impact of exposure to PMF at different frequencies, periods against *S. aureus*.

The Gram-positive candidate *S. aureus* yielded highly localized responses, highlighting the physical impact of cell wall structural differences:

- MIC endpoints and synergism: Synergistic interactions (FICI ≤ 0.5) were highly discrete, occurring only at two isolated parameters: PMF exposure at 20 Hz for 60 min, and PEF exposure at 6 Hz for 60 min (Fig. 10).
- Antibiogram screening: PEF exposure consistently enhanced antibiotic efficacy, leading to a measurable increase in the diameters of inhibition zones, peaking at 20 Hz for 20 min. In contrast, PMF generally decreased zone diameters, signaling reduced antibiotic efficacy. However, a highly selective exception was observed with AZM, which unexpectedly transitioned from resistant to sensitive following PMF treatment.
- Virulence profile: The virulence profile of *S. aureus* remained robust; the isolate maintained its characteristic $\beta$-hemolytic activity completely unaltered across all PEF and PMF frequencies and durations.
- Biofilm inhibition: Comparative analysis revealed that *S. aureus* was significantly less susceptible to biofilm disruption than the Gram-negative strains (Figs. 11 and 12). PMF exposure triggered the weakest overall response, peaking at just 45% inhibition (6 Hz, 20 min) and dropping as low as 4% (20 Hz, 60 min). PEF treatments were notably more effective, yielding a maximum inhibition of 60% (0.7 Hz, 20 min) and a minimum threshold of 29% (6 Hz, 60 min).

## Discussion

The selection of *S. aureus*, *E. coli*, and *P. aeruginosa* was strategically designed to evaluate whether the observed bioelectric phenomena are universal or dependent on specific cell envelope architectures. *S. aureus* was utilized as the most standard for Gram-positive research. Its monoderm architecture characterized by a thick (20–80 nm), robust peptidoglycan layer lacking an outer membrane serves as a sufficient model to study how electromagnetic fields (EMF) penetrate dense, porous cell walls^52^. While both *E. coli* and *P. aeruginosa* are Gram-negative, they were both included to address the massive disparity in their physical and chemical resistance profiles. *E. coli* represents a standard, relatively permeable model where drug entry occurs via common porin channels^53^. In contrast, *P. aeruginosa* serves as a fortified model; its outer membrane is 10 to 100 times less permeable than that of *E. coli*^54^. This dual strain approach allowed us to determine if EMF treatment can facilitate drug delivery in both standard and highly resistant physiological environments. The two Gram-negative strains offer distinct insights into antibiotic-field interactions. While *E. coli* resistance is primarily enzymatic (mutations in DNA gyrase), *P. aeruginosa* utilizes aggressive active ejection via efflux pumps such as MexAB-OprM system^55^. By comparing them, we could observe if EMF primarily interferes with passive diffusion (*E. coli*) or active ejection (*P. aeruginosa*).

The transition in the physical profiles of the nanoparticles post-loading specifically the increase in particle size from 122 ± 1.2 nm to 160 ± 2.6 nm and the shift in PDI from 0.22 ± 0.04 to 0.34 ± 0.06 offers distinct functional advantages across multiple biophysical parameters. From a structural standpoint, the enlargement from 122 nm to 160 nm directly reflects successful core drug loading, facilitating an exceptionally high encapsulation efficiency (97 ± 0.57%) by creating an optimal volume-to-core ratio within the cross-linked chitosan-TPP matrix. Biologically, a particle size of 160 nm is highly advantageous for controlled release behavior; it avoids the rapid burst release typical of smaller nanostructures by lengthening the diffusion pathway of ciprofloxacin out of the polymer core, ensuring sustained delivery at the infection site. Furthermore, despite the moderate increase in PDI to 0.34, the system maintains a zeta potential of + 40 ± 2.51 mV, which provides strong electrostatic repulsion to preserve long-term colloidal stability and prevent spontaneous aggregation in solution. This controlled size profile is perfectly optimized for biomedical applications; it falls within the ideal window to ensure high antibacterial efficacy by maximizing localized drug retention while being large enough to limit non-specific mammalian cellular uptake, thus minimizing systemic toxicity.

Importantly, the particle size obtained in this study, along with the observed positive zeta potential, aligns closely with the characteristics of previously documented antimicrobial nanomaterials. These nanomaterials have demonstrated a significant ability to interact with the surfaces of bacterial cells, leading to increased effectiveness in combating resistant pathogens^11^. This enhanced interaction is crucial for improving antimicrobial efficacy, as it allows for better penetration and disruption of bacterial cell membranes, ultimately resulting in a more potent antimicrobial response^11,12^. When evaluated against broader microbiological benchmarks, these characterization metrics and the resulting MIC reductions parallel recent nano-enabled antimicrobial strategies targeted at multidrug-resistant (MDR) ESKAPE pathogens. Previous studies optimizing metal oxide and polymeric nanoparticles against highly defensive strains like *P. aeruginosa* and *S. aureus* demonstrate that controlling the nanostructure’s physical presentation is a prerequisite to overcoming adaptive envelope barriers^9,56^. By achieving a highly uniform distribution that sits comfortably within these established structural windows, our system successfully mirrors the baseline efficacy seen in state-of-the-art ESKAPE-targeted platforms, validating its deployment against highly resistant clinical isolates.

The primary mechanism observed across all species is the bioelectric effect, driven by the application of PEF. This phenomenon describes a multi-stage interaction where the external field actively modifies both the structural integrity of the biofilm matrix and the physiological state of the bacterial cells^51,57^. PEF acts as a physical “permeabilizer,” inducing dielectric breakdown and the formation of transient, nanometer-sized pores within the lipid bilayer^58^. This electromechanical stress destabilizes the transmembrane potential (Vm), which can be modeled by the Goldman Hodgkin Katz (GHK) Voltage Equation. By increasing effective porin size and membrane permeability, PEF allows Cipro-C-NPs to bypass physical defenses and reach intracellular targets specifically DNA gyrase more efficiently^59^. Nevertheless, it is crucial to recognize that the overall antimicrobial mechanisms associated with these advanced nanoparticles are highly complex and arise from multiple interconnected factors beyond physical pore formation. In addition to improved membrane permeability, antimicrobial nanomaterials can exert their cytotoxic effects through the generation of ROS that induce oxidative stress, the direct chemical destabilization of lipid bilayers, the inhibition of quorum sensing networks, structural damage to vital intracellular proteins, and the targeted down-regulation or suppression of biofilm-related genes^60^. This multi-factorial approach ensures that physical field effects and polymer-based delivery work in tandem to systematically dismantle bacterial survival pathways^9^.

A recurring pattern in this study is the existence of highly specific biological frequency windows^61,62^. While PEF provides direct physical disruption, PMF operate primarily via a resonance mechanism. In *E. coli*, synergy was restricted to 0.7 Hz. In *S. aureus*, synergy was observed at 20 Hz (PMF) and 6 Hz (PEF). This suggests that specific frequencies match the natural oscillation of membrane-bound proteins, ion pumps, or teichoic acids^63^. In some instances, such as with *E. coli* and *P. aeruginosa*, PMF induced resonance may over stimulate active efflux systems (AcrAB-TolC pump), physically expelling antibiotics before they reach their target.

The variability in antimicrobial response is rooted in the fundamental differences in bacterial architecture in a way that it shows Gram-Negative barrier or Gram-Positive resilience. *E. coli* possesses a permeable outer membrane that is highly susceptible to pore formation. In contrast, *P. aeruginosa* is 10 to 100 times less permeable and produces alginate exopolysaccharides^54^. This thick dielectric environment can act as a physical shield, requiring specific resonance (20 Hz) to reach the threshold necessary to interfere with intracellular targets^62^. On the other hand, the thick, cross linked peptidoglycan layer of *S. aureus* acts as a protective buffer^52^. Electromagnetic fields must induce dielectric polarization within this dense matrix to loosen the molecular sieve and facilitate nanoparticle influx^32^.

Electromagnetic stress disrupts the collective behavior of bacterial populations through quorum quenching and virulence modulation. PMF induced magnetokinetic stress likely disrupts the ionic environments or conformational stability of receptor proteins (LasR/RhlR) required for signal transduction^63^. This silencing of communication was most evident in *P. aeruginosa*, where PMF exposure completely abolished the production of the redox-active virulence factor pyocyanin. This robust suppression of key virulence traits has profound implications for reducing bacterial pathogenicity and mitigating the development of resistance. By non-invasively disrupting the production of pyocyanin and down-regulating signaling networks, the treatment actively disarms the pathogen’s biochemical weaponry without immediately threatening its basic survival. This specific attenuation limits the selective evolutionary pressure that typically drives mutations and adaptive resistance during aggressive, high-dose antibiotic regimens. Furthermore, neutralizing these virulence factors directly impairs the structural stabilization of the extracellular matrix, leaving the bacterial population highly vulnerable to host immune clearance and concurrent antibiotic action.

Similarly, the reduction of N-acyl homoserine lactone (AHL) signaling molecules prevents biofilms from reaching structural maturity^64^. Disrupting biofilms is a crucial therapeutic target since mature biofilms shield bacterial communities from antibiotics and immune responses, acting as a primary driver of phenotypic antimicrobial resistance^56^. Antimicrobials derived from natural products and nanomaterials have demonstrated significant potential in preventing the establishment and maturation of biofilms^11^. The consistent reduction in biofilm formation across most conditions highlights a fitness trade off. The metabolic cost of maintaining robust adaptive resistance mechanisms and a thick peptidoglycan layer under physical stress diverts the energy budget away from biofilm-associated protein synthesis^65^. Furthermore, PEF-induced structural perturbations of surface appendages like fimbriae and pili prevent the transition from a planktonic state to a sessile biofilm community. However, the wide range of biofilm inhibition observed across different exposure regimes such as in *S. aureus*, where responses varied from a negligible 4% to a robust 45% inhibition highlights the highly non-linear nature of bioelectromagnetic interactions. Rather than indicating stochastic instability or poor reproducibility, this high sensitivity to specific combinations of frequency and duration reflects the existence of narrow, discrete “biophysical windows” of efficacy. In a clinical or translational setting, this underscores that physical field treatments cannot be applied as broad, generalized exposures. Instead, they require strict protocol standardization and precise parameter lock-in. Just as specific dosages are vital for pharmaceuticals, identifying and fixing the exact therapeutic “sweet spots” (such as the specific synergistic frequencies identified in this screening) is an absolute prerequisite to achieving reproducible clinical outcomes.

This non-linear behavior is further illustrated by the notable observation in Fig. 9, where Chitosan NPs demonstrated a superior standalone antibiofilm effect compared to the drug-loaded Cipro-C-NPs. This phenomenon is driven by the intrinsic, highly polycationic nature of pure chitosan, which possesses a high density of unoccupied protonated amino groups (NH_3_^+^). These positive charges interact directly and aggressively with the negatively charged components of the bacterial extracellular polymeric substance (EPS) matrix such as alginate and extracellular DNA (eDNA) causing severe electrostatic destabilization and structural collapse of the biofilm shield. Conversely, when chitosan is loaded with Ciprofloxacin, a portion of these active cationic amino groups undergoes partial neutralization through ionic interactions with the drug molecules, as confirmed by the lower baseline zeta potential observed during characterization. This charge utilization, combined with modified drug release kinetics within the dense matrix, slightly dampens the immediate, surface-mediated electrostatic disruption of the biofilm compared to the fully exposed positive charge of the nanoparticles.

To understand the molecular vulnerability of the delivery system to external fields, the fundamental nature of the drug-polymer matrix must be examined. The exceptionally high encapsulation efficiency observed is dictated by strong electrostatic and ionic interactions between the protonated primary amino groups (NH_3_^+^) along the chitosan backbone and the deprotonated carboxylate groups (COO-) of the zwitterionic ciprofloxacin molecule. When exposed to extremely low-frequency electromagnetic fields, this localized electrostatic equilibrium is actively perturbed. The oscillating physical fields exert localized Lorentz forces and induce dielectric polarization across these highly charged functional groups. This electromagnetic stress generates microscopic structural torque and molecular vibrations that temporarily weaken the ionic attraction and hydrogen bonding network holding the drug within the polymeric mesh. Consequently, exposure to physical fields can directly modulate the structural stability of the matrix, altering drug-binding efficiency and driving accelerated or altered drug-release kinetics depending on the specific frequency applied. Under synergistic parameters, this field-induced disruption acts as a physical trigger that commandingly accelerates drug liberation to overwhelm bacterial defenses; conversely, under poorly calibrated regimes, the disruption of this internal electrostatic balance leads to the surface charge rearrangements and subsequent colloidal aggregation observed elsewhere in our study.

Additionally, the reduced efficacy and in specific instances, true antagonism (such as PMF exposure at 0.7 Hz for 20 min against *P. aeruginosa*) points directly to field-induced alterations in the nanomaterial’s physical properties. The applied electromagnetic and electric fields disrupt the delicate equilibrium of the colloidal suspension, causing a rearrangement of surface charges that lowers the zeta potential of the nanoparticles^65^. This reduction in zeta potential diminishes the critical electrostatic repulsion keeping the particles isolated^66,67^. Once this potential falls below a stable threshold, attractive forces dominate, forcing the independent nanoparticles to lock together into large, multi-particle colloidal aggregates. This physical clumping creates an immediate biological bottleneck. While single, well-dispersed nanoparticles (~ 160 nm) can effectively interact with bacterial envelopes, these large, field-induced aggregates suffer from a severely reduced surface-area-to-volume ratio, limiting their overall contact efficiency with bacterial membranes. Biomedically, this dramatic reduction in active surface area alters the spatial presentation of the protonated amino groups (NH_3_^+^), sterically hindering their capacity to achieve multi-point electrostatic docking with the negatively charged bacterial lipopolysaccharides (Gram-negative) or teichoic acids (Gram-positive)^52–54^. Consequently, membrane adhesion is severely compromised, preventing the localized destabilization of the bacterial envelope. Furthermore, cellular uptake via passive membrane trans-location or porin-mediated influx is completely halted, as these macro-aggregates face strict structural size exclusion by exceeding the strict dimensions of outer membrane porin channels and the cross-linked peptidoglycan meshwork^67,68^. Trapped extracellularly, the nanoparticles cannot execute intracellular drug delivery or disrupt cytoplasmic machinery, which drastically lowers their localized antibacterial activity and drives up Minimum Inhibitory Concentrations (MICs). Far from a random anomaly, this reveals that poorly calibrated frequencies or exposure times can inadvertently fortify bacterial defenses by transforming an advanced delivery system into an excluded physical mass. While this field induced colloidal aggregation limits efficacy under specific exposure regimes, it should not be viewed as an inherent flaw in the system, but rather as evidence of a highly tunable, parameter-dependent biophysical switch. In translational applications, this dual behavior highlights a critical optimization window: poorly calibrated frequencies or intensities can inadvertently trigger aggregation and fortify bacterial defenses, whereas precisely optimized parameters can maintain or enhance colloidal stability to maximize membrane penetration. Rather than undermining the utility of the approach, these findings demonstrate that electromagnetic fields offer a non-invasive mechanism to actively modulate nanomaterial kinetics either suppressing or accelerating drug delivery based strictly on physical parameter tuning. Ultimately, these findings carry significant broader implications for addressing the escalating global antimicrobial resistance (AMR) crisis. As conventional monotherapies continue to fail against fortified MDR pathogens, advanced nano-enabled formulations coupled with non-invasive biophysical fields offer a highly promising alternative or complement to the clinical pipeline. By operating through a synchronized, multi-targeted network where physical field forces destabilize physical barriers and the polymeric nanocarriers optimize localized drug delivery this approach minimizes the therapeutic dosages required to achieve eradication. Moving away from traditional high-dose pharmaceutical approaches toward precision-targeted, physics-assisted antimicrobial therapies provides a sustainable framework to preserve existing antibiotics, lower systemic toxicity, and elevate the translational significance of alternative nanomedicine platforms.

## Conclusion

This study suggests that ELF PEMW exposure can function as a biophysical antagonist rather than a synergist for Cipro-loaded chitosan nanoparticles under specific conditions, fundamentally altering the expected drug-pathogen interaction. Based on our exploratory findings, we propose a biophysical model wherein the magnetic field induces membrane hyperpolarization, potentially increasing the magnitude of the transmembrane potential. This electrical shift is hypothesized to restrict porin-mediated transport, effectively bottlenecking the primary entry route for the antibiotic. Simultaneously, the electromagnetic field appears to destabilize the colloidal state of the delivery system by reducing the zeta-potential of the chitosan nanoparticles. This reduction in electrostatic repulsion likely leads to nanoparticle aggregation, lowering the surface area-to-volume ratio and hindering the particles’ ability to dock with bacterial surfaces. The resulting size exclusion effect, where large aggregates can no longer navigate the narrow dimensions of bacterial porins, provides a plausible physical explanation for the observed increase in MICs. Furthermore, the divergent responses of *P. aeruginosa* and *E. coli* suggest that electromagnetic susceptibility is highly sensitive to species-specific membrane capacitance and porin density, reflecting a complex frequency-response relationship. Ultimately, these findings reveal a critical bio-electromagnetic trade-off; while physical forces offer a novel means of cellular modulation, poorly tuned parameters can inadvertently fortify bacterial defenses and reduce drug bioavailability. While these inferred biophysical mechanisms require direct real-time electrophysiological confirmation in future studies, they provide a vital conceptual framework. To successfully translate this framework into a clinical reality, future research must simultaneously address the physical constraints of human anatomy by evaluating tissue-penetration dynamics across varying depths and validating host biocompatibility to eliminate off-target effects. Moving toward both the direct electrophysiological mapping of efflux pump kinetics and membrane potential to achieve a precision-targeted, physics-assisted antimicrobial strategy. While these exploratory findings are qualitative and require future statistical validation using expanded biological replicates, they provide an essential first step into the unexplored area of bioelectromagnetic synergy.

## Scope, limitations and future directions

The complex, multi-factorial design of this study encompassing three antibacterial agents, three bacterial strains, two physical fields (PMF and PEF), multiple frequencies, and varying exposure durations necessitated an exploratory approach. Because this combinatorial parameter space is exceptionally large, many experimental conditions were evaluated using a single replicate (*n* = 1) or a single clinical isolate per species to serve as a high-throughput preliminary screen. Consequently, the findings presented herein are descriptive and qualitative; no inferential statistical claims can be made regarding the significance or magnitude of the observed effects.

Nevertheless, the scientific value of this research lies in its novel mapping of the intricate interplay between nanomaterials, antibiotics, and non-ionizing electromagnetic fields. These preliminary data provide a critical proof-of-concept, establishing a roadmap for future, statistically powered research. Follow-up investigations will focus on the most promising parameter sets identified here (6 Hz frequencies or 20-minute exposures) to establish definitive statistical confirmation, determine dose-response relationships, and elucidate the underlying molecular mechanisms. Additionally, a key limitation of the current in vitro framework is the lack of in vivo validation. Translating these findings to a clinical setting requires addressing how complex physical fields penetrate heterogeneous human tissues to reach deep-seated infection sites. Future translational research must evaluate field attenuation across different tissue depths, optimize coil geometries for targeted delivery, and conduct rigorous safety assessments to ensure that the chosen electromagnetic parameters do not induce off-target electrophysiological or thermal effects in host tissues.

## Supplementary Information

Below is the link to the electronic supplementary material.

Supplementary Material 1

## Data Availability

All data supporting the findings of this study are available within the paper.
