# Supplementary material for: Evaluation of the synergistic and antagonistic antibacterial effects of pulsed electromagnetic fields combined with ciprofloxacin and nanochitosan
Source: Sci Rep. 2026 Jul 23;16:23063. doi: 10.1038/s41598-026-63235-2 (PMC13392081; doi:10.1038/s41598-026-63235-2)
Supplement: Supplementary file 1 — Supplementary Material 1 [file 41598_2026_63235_MOESM1_ESM.pdf]

Supplementary Appendix

This supporting document contains the raw phenotypic verification data, original disc diffusion plates, and biochemical assay records validating the field-dependent changes described in the main text.

1. Phenotypic Characterization and Virulence Profiles
   a- Baseline Hemolytic Activity Assessments

Hemolysin production is a key pathogenic determinant involved in cell envelope degradation and host tissue damage. To determine whether exposure to external physical fields alters baseline hemolytic expression, isolates were cultured on blood agar plates under both control and maximum field exposure conditions.

The images shown in Figures S1 and 2 indicate that both *Escherichia coli* and *Staphylococcus aureus* consistently maintained their characteristic hemolytic phenotypes completely unaltered following maximum exposure to pulsed electric fields (PEF) and pulsed magnetic fields (PMF) across all frequencies (0.7 Hz, 6 Hz, and 20 Hz). *S. aureus* maintained full, clear zones of erythrocyte lysis (β-hemolysis). *E. coli* maintained its baseline profile, confirming that the physical fields modulate susceptibility without fundamentally modifying or muting native hemolytic virulence genes.

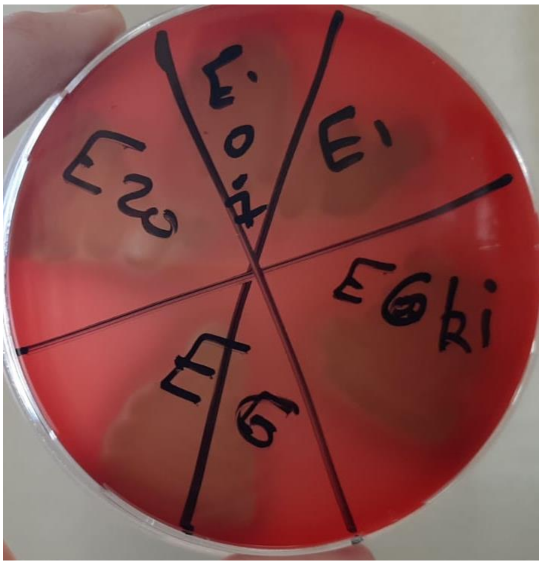

Figure S1. Representative blood agar plate showing stable, unattenuated β -hemolytic activity of *Escherichia coli* clinical isolates under baseline control and exposed conditions.

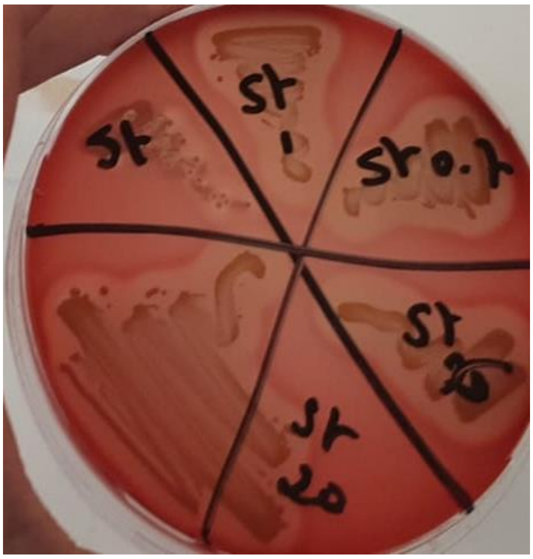

Figure S2. Representative blood agar plate showing persistent, full β -hemolytic zones around *Staphylococcus aureus* colonies following exposure to maximum physical field parameters.

b- Modulation of Pyocyanin Production in *Pseudomonas aeruginosa*

Pyocyanin is a prominent redox-active phenazine toxin secreted by *P. aeruginosa* that drives oxidative stress during infection as shown in Figures S3 and S4. The application of external physical fields revealed a distinct, parameter-dependent divergence in pigment expression. Exposure to increasing frequencies of PMF triggered a noticeable, dose-dependent decrease in blue-green pyocyanin production. At 20 Hz exposure, the pigment expression was highly suppressed, transitioning the culture medium to a pale-yellow state, confirming successful phenotypic virulence mitigation. In contrast to the magnetic field, exposure to PEF generated an opposing physiological response, visually intensifying the characteristic blue-green pyocyanin pigment in the medium. The images presented here are representative samples selected from the complete experimental dataset to visually illustrate the macroscopic changes observed. While these photographs serve primarily for qualitative demonstration, they are provided to ensure full experimental transparency and to assist the reader in visualizing the distinct phenotypic variations.

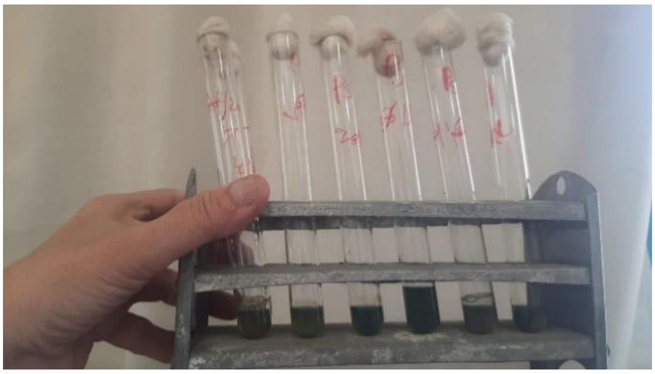

Figure S3. Visual changes in pyocyanin pigment suppression and modulation in *Pseudomonas aeruginosa* liquid cultures following physical field exposure. Test tubes containing clinical isolates are arranged from left to right in order of increasing frequency and exposure duration: 0.7 Hz (20 min), 6 Hz (20 min), 20 Hz (20 min), 0.7 Hz (60 min), 6 Hz (60 min), and 20 Hz (60 min). Distinct color transitions in the media follow a specific pattern correlated with the field parameters.

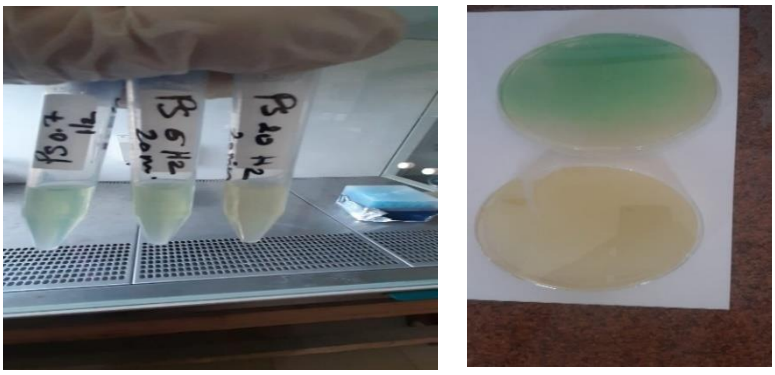

Figure S4. Qualitative tracking of pyocyanin pigment suppression on *Pseudomonas aeruginosa* due to PMF exposure. (A) tubes containing cultures exposed to PMF for 60 min at varying frequencies (0.7 Hz, 6 Hz, and 20 Hz, left to right), demonstrating a frequency-dependent coloration pattern. (B) Pseudomonas agar plates streaked with a loopful of inoculated broth from the control (unexposed) strain (top) and the PMF-exposed strain (bottom). A drastic decrease in the intensity of the blue-green pyocyanin pigment is clearly visible in the exposed sample compared to the control.

2. Antibiogram and Disc Diffusion Variations

The structural and physiological adaptations induced by external fields directly altered the diffusion dynamics and effectiveness of conventional antibiotic discs. The images presented here are representative samples selected from the complete experimental dataset to visually illustrate the zones of inhibition. These plates include standard antibiotic discs to assist the reader in visualizing the comparative treatment effects. They are provided as illustrative qualitative aids to ensure full experimental transparency.

a- *Escherichia coli* Antibiogram Shifts

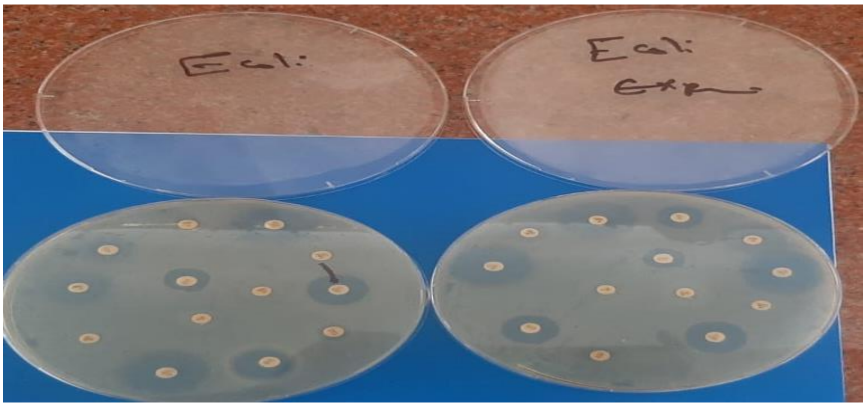

Figure S5. Modification of disc diffusion zones of inhibition for *Escherichia coli* following exposure to a PMF of 6 Hz for 20 minutes, illustrating parameter-dependent changes in membrane permeability and drug susceptibility.

b- *Pseudomonas aeruginosa* Antibiogram Shifts

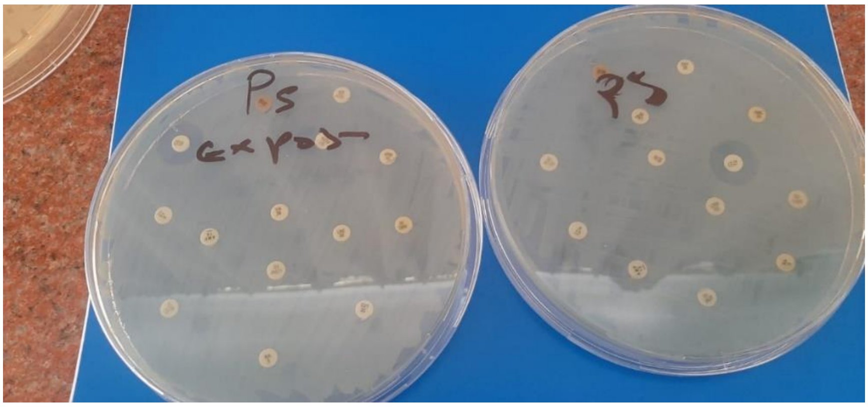

Figure S6. Disc diffusion profile comparing the original unexposed *Pseudomonas aeruginosa* strain with the strain subjected to a 0.7 Hz PMF regime, highlighting shifts in antibiotic susceptibility patterns.

c- *Staphylococcus aureus* Antibiogram Shifts

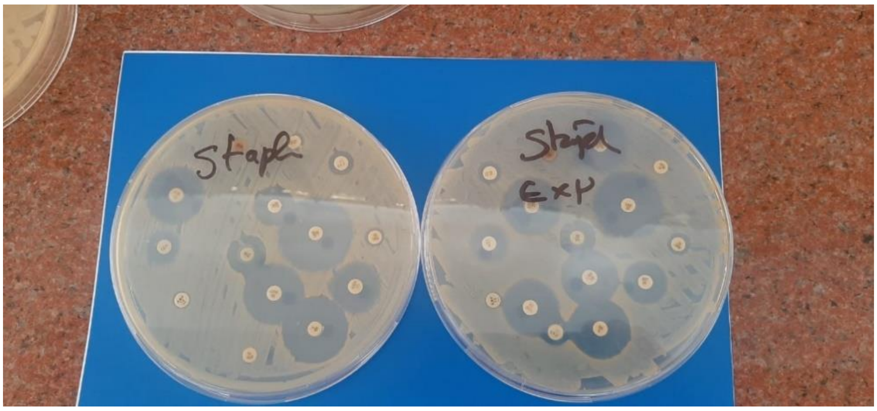

Figure S7. Disc diffusion assay of *Staphylococcus aureus* clinical isolates following a 20 Hz PMF exposure sequence, providing visual validation for the zone diameters compiled in the main text.

Antibiogram Raw Zone Potentials (mm)

The data presented in this table represents selected raw values from individual experimental runs, intended solely to illustrate the qualitative patterns of antibiotic susceptibility and resistance following exposure. These values have not been averaged and are not accompanied by statistical calculations. They are provided as tentative, indicative measures to serve as a qualitative evaluation of the treatment trends.

Escherichia coli																			
**PMF Exposure**	CI 10	TE 30	ATM 10	LNZ 30	AMC 30	SXT 25	VA 30	LOM 10	RA 5	FAD 10	TOB 10	AMP 10	S 10	AZM 15	COT 25	OFX 5	GEN 10	CRO	CXM
Unexposed	15	—	25	—	—	—	—	30	—	NA	27	—	17	—	—	27	—	25	24
0.7 Hz / 20 min	14	—	—	—	—	—	—	28	—	NA	27	—	13	—	—	—	—	—	22
0.7 Hz / 60 min	15	—	—	—	—	—	—	26	—	NA	26	—	11	—	—	—	—	—	20
6.0 Hz / 20 min	15	—	—	—	—	—	—	25	—	NA	27	—	10	—	—	—	—	—	19
6.0 Hz / 60 min	15	—	—	—	—	—	—	25	—	NA	26	—	9	—	—	—	—	—	—
20 Hz / 20 min	14	—	—	—	—	—	—	25	—	NA	26	—	9	—	—	—	—	—	—
20 Hz / 60 min	15	—	—	—	—	—	—	23	—	NA	26	—	8	—	—	—	—	—	—

Pseudomonas aeruginosa																			
**PMF Exposure**	CI 10	TE 30	ATM 10	LNZ 30	AMC 30	SXT 25	VA 30	LOM 10	RA 5	FAD 10	TOB 10	AMP 10	S 10	AZM 15	COT 25	OFX 5	GEN 10	CRO	CXM
Unexposed	16	—	—	—	—	—	—	—	—	NA	—	—	—	—	—	—	—	—	—
0.7 Hz / 20 min	15	—	—	—	—	—	—	—	—	NA	—	—	—	—	—	—	—	—	—
0.7 Hz / 60 min	15	—	—	—	—	—	—	—	—	NA	—	—	—	—	—	—	—	—	—
6.0 Hz / 20 min	14	—	—	—	—	—	—	—	—	NA	—	—	—	—	—	—	—	—	—
6.0 Hz / 60 min	14	—	—	—	—	—	—	—	—	NA	—	—	—	—	—	—	—	—	—
20 Hz / 20 min	15	—	—	—	—	—	—	—	—	NA	—	—	—	—	—	—	—	—	—
20 Hz / 60 min	15	—	—	—	—	—	—	—	—	NA	—	—	—	—	—	—	—	—	—

Staphylococcus aureus																			
**PMF Exposure**	CI 10	TE 30	ATM 10	LNZ 30	AMC 30	SXT 25	VA 30	LOM 10	RA 5	FAD 10	TOB 10	AMP 10	S 10	AZM 15	COT 25	OFX 5	GEN 10	CRO	CXM
Unexposed	—	—	—	34	9	20	17	29	30	14	28	—	15	—	—	27	17	—	22
0.7 Hz / 20 min	—	—	—	33	9	20	17	27	27	14	28	—	13	—	—	27	15	—	20
0.7 Hz / 60 min	—	—	—	32	9	19	17	25	26	12	26	—	13	22	23	25	15	—	20
6.0 Hz / 20 min	—	—	—	32	9	20	16	24	26	11	25	—	13	24	23	24	15	—	19
6.0 Hz / 60 min	—	—	—	33	9	20	17	25	27	11	25	—	13	23	24	25	15	—	20
20 Hz / 20 min	—	—	—	28	7	16	14	22	22	10	23	—	11	24	22	22	13	—	17
20 Hz / 60 min	—	—	—	26	6	15	13	20	20	9	21	—	10	22	21	20	11	—	15

Escherichia coli																			
**PEF Exposure**	CI 10	TE 30	ATM 10	LNZ 30	AMC 30	SXT 25	VA 30	LOM 10	RA 5	FAD 10	TOB 10	AMP 10	S 10	AZM 15	COT 25	OFX 5	GEN 10	CRO	CXM
Unexposed	15	—	25	—	—	—	—	30	—	NA	27	—	17	—	—	27	—	25	24
0.7 Hz / 20 min	20	—	26	—	—	—	—	32	—	NA	29	—	17	—	—	28	20	29	26
0.7 Hz / 60 min	21	12	30	—	—	—	—	30	—	NA	25	—	16	—	30	25	21	23	30
6.0 Hz / 20 min	18	10	24	—	—	—	—	21	—	NA	24	—	16	—	20	23	20	22	22
6.0 Hz / 60 min	19	—	23	—	—	—	—	20	—	NA	24	—	17	—	22	22	21	23	20
20 Hz / 20 min	18	—	22	—	—	—	—	20	—	NA	25	—	16	—	23	23	21	24	21
20 Hz / 60 min	20	—	23	—	—	—	—	21	—	NA	24	—	16	—	22	22	21	22	25

Pseudomonas aeruginosa																			
**PEF Exposure**	CI 10	TE 30	ATM 10	LNZ 30	AMC 30	SXT 25	VA 30	LOM 10	RA 5	FAD 10	TOB 10	AMP 10	S 10	AZM 15	COT 25	OFX 5	GEN 10	CRO	CXM
Unexposed	16	—	—	—	—	—	—	—	—	NA	—	—	—	—	—	—	—	—	—
0.7 Hz / 20 min	20	—	14	—	—	—	—	—	—	NA	—	—	—	11	—	—	—	—	—
0.7 Hz / 60 min	23	—	16	—	—	—	—	—	—	NA	—	—	—	13	—	—	—	—	—
6.0 Hz / 20 min	22	—	12	—	—	—	—	—	—	NA	—	—	—	11	—	—	—	—	—
6.0 Hz / 60 min	21	—	11	—	—	—	—	—	—	NA	—	—	—	10	—	—	—	—	—
20 Hz / 20 min	21	—	15	—	—	—	—	—	—	NA	—	—	—	12	—	—	—	—	—
20 Hz / 60 min	21	—	14	—	—	—	—	—	—	NA	—	—	—	12	—	—	—	—	—

Staphylococcus aureus																			
**PEF Exposure**	CI 10	TE 30	ATM 10	LNZ 30	AMC 30	SXT 25	VA 30	LOM 10	RA 5	FAD 10	TOB 10	AMP 10	S 10	AZM 15	COT 25	OFX 5	GEN 10	CRO	CXM
Unexposed	—	—	—	34	9	20	17	27	30	14	28	—	15	—	—	29	17	—	22
0.7 Hz / 20 min	—	11	—	40	15	20	20	28	30	14	30	—	15	11	28	30	17	—	22
0.7 Hz / 60 min	—	11	—	35	13	20	19	22	33	15	28	—	16	—	29	30	18	—	30
6.0 Hz / 20 min	—	11	—	34	13	19	20	23	30	14	28	—	15	—	28	28	18	—	29
6.0 Hz / 60 min	—	11	—	34	13	20	20	22	33	14	28	—	16	—	29	28	17	—	30
20 Hz / 20 min	—	15	—	41	15	22	20	29	35	16	31	—	18	—	32	32	20	—	31
20 Hz / 60 min	—	15	—	35	13	19	20	24	30	15	28	—	16	—	30	25	18	—	29
